# Transforming growth factor β induced as a novel secreted immune checkpoint counterinhibiting human tumor-associated T cells

**DOI:** 10.1136/jitc-2025-012668

**Published:** 2026-04-02

**Authors:** Maria Manuela Rosado, Eleonora Timperi, Ombretta Melaiu, Sara Vitale, Sophie Bachy, Alessio Grimaldi, Mariachiara Corrado, Vanessa Mancini, Gabriela Leonti, Filippo Conti, Ilenia Cammarata, Ivana Celardo, Stephanie Kucykowicz, Gloryanne Aidoo-Micah, Paula Gragera, Daniele Accapezzato, Maria Del Ben, Valentina D’Oria, Cristiano De Stefanis, Laura Forcina, Fabrizio Frattaroli, Andrea Picchetto, Massimo Chiaretti, Giancarlo D’Ambrosio, Giuseppe Giannini, Francesca Belardinilli, Andrea Scarinci, Gian Luca Grazi, Antonio Musarò, Mala K Maini, Micol E Fiori, Doriana Fruci, Anca Hennino, Vincenzo Barnaba

**Affiliations:** 1 Department of Research, Advanced Diagnostic and Technological Innovation Translational Research Functional Departmental Area, Regina Elena Institute, Roma, Italy; 2 Department of Internal Clinical Sciences, Anesthesiology and Cardiovascular Sciences, Sapienza University of Rome, Rome, Italy; 3 Università Cattolica del Sacro Cuore Facoltà di Medicina e Chirurgia, Rome, Italy; 4 Department of Paediatric Haematology/Oncology and Cell and Gene Therapy, Bambino Gesu Pediatric Hospital IRCCS, Rome, Italy; 5 Department of Clinical Science and Translational Medicine, “Tor Vergata” University of Rome, Rome, Italy; 6 Istituto Superiore di Sanità, Rome, Italy; 7 StromaCare Co, Lyon, France; 8 Department of Molecular Medicine, Sapienza University of Rome, Roma, Italy; 9 Institute of Immunity and Transplantation, Division of Infection and Immunity, UCL, London, England; 10 Confocal Microscopy Core Facility, Bambino Gesu Pediatric Hospital, Roma, Italy; 11 Flow Cytometry and Histology Core Facilities, Bambino Gesu Pediatric Hospital, Roma, Italy; 12 DAHFMO-Unit of Histology and Medical Embryology, Sapienza University of Rome, Rome, Italy; 13 Istituto Pasteur Italia – Fondazione Cenci Bolognetti, Istituto Pasteur Italia, Rome, Italy; 14 Department of General and Specialistic Surgery, Sapienza University of Rome, Rome, Italy; 15 Hepato-Pancreato-Biliary Surgery Unit, IRCCS Regina Elena National Cancer Institute, Rome, Italy; 16 Cancer Research Centre Lyon (CRCL), Lyon, France

**Keywords:** Colorectal Cancer, Hepatocellular Carcinoma, Immune Checkpoint Inhibitor, Immunosuppression, T-Lymphocytes

## Abstract

**Background:**

We investigated the hypothesis that transforming growth factor β induced (TGFBI), an extracellular matrix protein secreted in the microenvironment of several tumors, can act as a secreted immune checkpoint (sIC) that contributes to the suppression of human antitumor T cell responses.

**Methods and results:**

Serum TGFBI concentrations, measured by ELISA, were significantly higher in patients with colorectal cancer (CRC) and hepatocellular carcinoma than in healthy individuals and associated with poor overall survival. Strikingly, multiparametric flow cytometry analyses revealed that TGFBI was abundantly expressed by tumor cells or monocytes, and by various lymphoid cell types—including CD4^+^ or CD8^+^ T cells (including tissue-resident memory T cells), B cells, and natural killer cells—in patients with cancer. Importantly, ex vivo TGFBI neutralization significantly enhanced CD4^+^ and CD8^+^ T cell activation and function. Freshly isolated TGFBI-expressing CD4^+^ or CD8^+^ T cells demonstrated a markedly improved capacity to differentiate into functional effector cells—characterized by the acquisition of tissue-homing phenotypes—following TGFBI blockade. These findings suggest that TGFBI can establish an autocrine immunosuppressive loop within T cells, thereby limiting their differentiation and function. These mechanistic observations were further supported by human CRC organoid-based experiments, where TGFBI blockade improved expansion and tumor cell killing by major histocompatibility complex class I-restricted cytotoxic T cells.

**Conclusions:**

Taken together, our data demonstrate that TGFBI acts as a sIC counter-regulating T cell activation, differentiation, and effector function, which can be restored by TGFBI blockade, with broad implications for novel immunotherapy strategies in solid tumors.

**WHAT IS ALREADY KNOWN ON THIS TOPIC**

- Transforming growth factor β induced (TGFBI) is an extracellular matrix protein secreted in the microenvironment of several solid tumors, and it has been associated with increased tumor survival, angiogenesis, metastasis, and immunosuppression; however, the specific effect by TGFBI on T cell functions remains underexplored.

**WHAT THIS STUDY ADDS**

- This study demonstrates that TGFBI is expressed by various tumor-associated immune cells, including CD4^+^ and CD8^+^ T cells, and can act as a novel secreted immune checkpoint that countersuppresses T effector cell differentiation in patients with colorectal cancer (CRC) and hepatocellular carcinoma.
- Notably, TGFBI neutralization significantly enhanced antitumor CD4^+^ and CD8^+^ T cell effector responses in patients ex vivo, as well as in organoid-based models.

**HOW THIS STUDY MIGHT AFFECT RESEARCH, PRACTICE, OR POLICY**

- This study supports TGFBI blockade as a potential therapeutic strategy to enhance immune effector-based cancer treatments, by increasing tumor-associated CD4^+^ and CD8^+^ T cell responses in patients with cancer.
- Furthermore, the expansion of tumor-specific cytotoxic T cells in CRC organoid/peripheral blood mononuclear cell cocultures following TGFBI blockade suggests this approach could be leveraged to develop adaptive immunotherapies selectively targeting tumor cells.

For the graphical abstract of this paper see figure 1.

**Figure 1:**
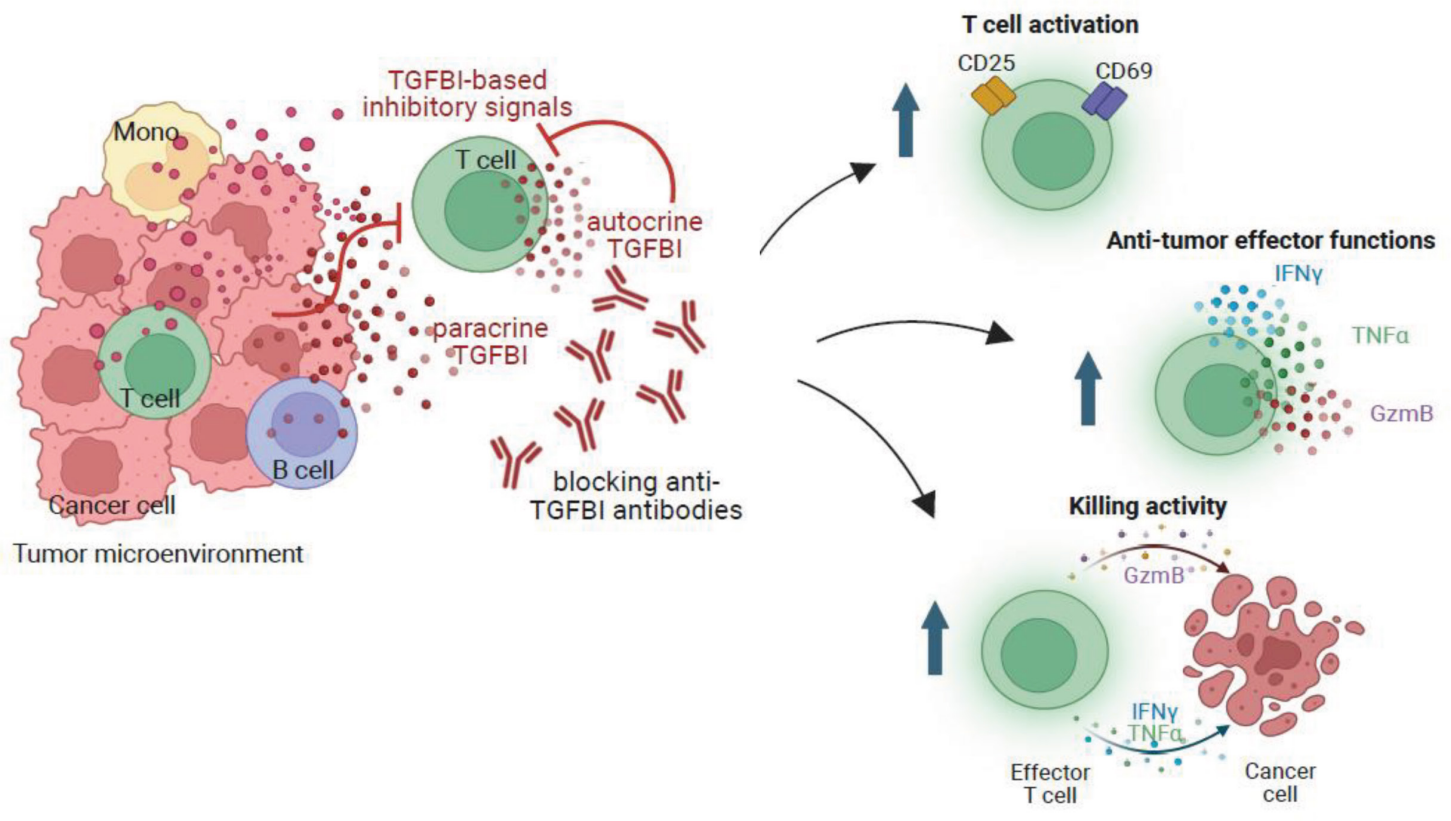
TGFBI as a novel secreted immune checkpoint counterinhibiting human tumor-associated T cells. In summary, this study proposes TGFBI blockade as a potential therapeutic strategy to enhance immune effector T cell functions in patients with cancer. GzmB, granzyme B; IFN, interferon; TGFBI, transforming growth factor β induced; TNF, tumor necrosis factor.

## Background

Tumor immune escape is commonly associated with tumor’s ability to deliver inhibitory signals to tumor-infiltrating lymphocytes (TILs), leading to T-cell exhaustion^1^ primarily through membrane-bound immune checkpoints (mICs).^2 3^ Monoclonal antibodies (mAbs) targeting these mICs—known as mIC inhibitors (mICIs)—have proven effective in restoring antitumor responses, resulting in remarkable clinical benefit in several metastatic cancers. These benefits are predominantly observed in ‘hot’ tumors—such as melanoma, non-small cell lung cancer, bladder, kidney, and head and neck cancers—which typically harbor high DNA instability due to defective mismatch repair (dMMR), resulting in a high mutational burden and a broad repertoire of neoantigens, along with high TIL frequency.^4–6^

However, current mICIs cause a partial or no remission in advanced tumors,^7^ as well as in the majority of the so-called ‘cold tumors’, which exhibit low somatic mutation rates and reduced TIL infiltration. Cold tumors include most breast, ovarian, prostate, pancreatic cancers, glioblastomas, as well as the majority of colorectal cancer (CRC) and hepatocellular carcinoma (HCC) cases, which are microsatellite stable (MSS) tumors and possess proficient MMR systems^8 9^ and are characterized by the prevalence of several cellular and molecular mechanisms of immunosuppression.^10^ Therefore, a main objective in cancer immunology is the development of novel strategies to convert cold tumors into immunologically active (hot) tumors.^11 12^

Secreted ICs (sICs)—such as Wnt3a, Wnt1, fibrinogen-like protein 1, IL-18BP, SCCA1/SERPINB3, netrin-1—have recently emerged as promising therapeutic targets in tumors resistant to conventional immune checkpoint inhibitor (ICI). Blocking of these sICs has been shown to restore immune cell migration, differentiation, and effector function, as well as inhibit tumor epithelial-to-mesenchymal transition.^13–16^ Targeting sICs may be particularly effective in cold tumors, as these soluble factors can disseminate throughout the tumor microenvironment (TME) and inhibit the activation and trafficking of competent effector T cells.

Transforming growth factor β induced (TGFBI), also known as βig-h3, is a 68 kDa (683 amino acids) extracellular matrix (ECM) protein induced by TGF-β and abundantly secreted into the TME of various solid tumors, due to its N-terminal secretory signal (aa 1–23).^17^ Through the Arg-Gly-Asp and FAS1 motifs, TGFBI interacts with ECM components—such as collagens I, II, IV, VI, and fibronectin—and α3β1, αVβ3, and αVβ5 integrins on cell surfaces,^17–19^ ultimately improving tumor survival, migration, invasion, tumorigenesis, angiogenesis, and metastasis.^20–23^ TGFBI has been proposed to function as a tumor suppressor in early tumors—by inhibiting proliferation and promoting apoptosis—and as a tumor promoter in advanced tumors, likely due to epigenetic silencing mechanisms being bypassed.^24^ High serum TGFBI levels correlate with poor survival in several cancers^22 25^ and are associated with increased infiltration of cancer-associated fibroblasts (CAFs), macrophages, myeloid-derived suppressor cells, and neutrophils, while inversely correlating with B cell, T follicular helper cell, and CD8^+^ T cell infiltration.^22 26 27^ Consistent with these findings, multiple studies in murine cancer models have shown that TGFBI is primarily produced by CAFs and macrophages and contributes to immune evasion by suppressing T cell responses—effects that can be reversed by TGFBI neutralization.^28–31^

Nevertheless, the direct immunological effects of TGFBI on antitumor T cell differentiation and functions remain incompletely defined. To address this gap, we provide evidence showing that TGFBI is expressed by stromal and myeloid cells,^32 33^ and by a wide range of lymphoid populations—including CD4^+^ T, CD8^+^ T, B, and natural killer (NK) cells—in patients with CRC and HCC. We show that TGFBI delivers counterinhibitory signals to tumor-associated T cells, and its neutralization improves differentiation and effector functions of peripheral and tumor-resident CD4^+^ and CD8^+^ T cells ex vivo, as well as the cytotoxic activity of tumor-specific T cells in CRC organoid models. These findings support the therapeutic potential of TGFBI blockade in reactivating antitumor immunity.

## Materials and methods

### Study population

Patients were recruited at Policlinico Umberto I, “Sapienza” University of Rome (Rome, Italy) and at the National Cancer Institute Regina Elena IRCCS (Rome, Italy) after being diagnosed for CRC (n=54) and HCC (n=62), respectively (table 1). Each patient donated serum, peripheral blood (PB), and when possible, non-tumor (NTUM) and tumor (TUM) tissues from the resection site after having signed the informed consent. Healthy donors (HDs) have been collected at Bambino Gesù Children’s Hospital (Rome, Italy) and included as controls. Collectively, 20 HDs have been used for in vitro stimulations and flow cytometry (FC) analysis. For the quantification of TGFBI serum concentration by ELISA, 20 healthy elderly (aged 69–82 years) undergoing therapy to control blood pressure were included. Study was approved by the Ethics Committee of Policlinico Umberto I (for CRC patients- study title “Identificazione di nuovi bersagli immunoterapeutici e di nuovi antigeni nel carcinoma del colon-retto”, Prot. Number 2065/15 and Prot. Number 0813/2021) and by Istituto Nazionale Tumori Regina Elena (Prot. Number 1137/18 for HCC patients) and all the procedures were performed in accordance with the ethical standards required by the 1975 Helsinki Convention. For the isolation of intrahepatic lymphocytes (IHLs), a different patient cohort was used, consisting of six patients undergoing surgical resections for HCC (n=3) and CRC-liver metastasis (n=3). All study participants gave written informed consent in accordance with the Declaration of Helsinki. Study approval was granted for the collection of patient samples from the Royal Free Hospital, London (Research Ethics Committee [REC] reference: 16/WA/0289, 11/WA/0077 or 11/H0720/4 [RIPCOLT clinical trial number 8191). Tissue samples obtained from patients undergoing surgical resection were collected by Tissue Access for Patient Benefit at the Royal Free Hospital, London and stored in University of Wisconsin solution at 4°C until processing.

**Table 1:** Clinical characteristics of patients enrolled in the study*

	Patients with CRC (n=54)	Patients with HCC (n=62)
Mean age (years, minimum–maximum)	71.5 (36–87)	70.8
Gender: female	28 (57%)	13 (21.05%)
Tumor site		Liver
Sigma	10	–
Colon	28	–
Recto	16	–
Grading		
G1	2 (3.7%)	1 (1.6%)
G2/G3	48 (88%)	55 (88.7%)
G4	4 (7.4%)	6 (9.6%)
Neoadjuvant therapy	5 (9.2%)	0
Microsatellite instability	1 (1.8%)	0
Cirrhosis	–	30 (52.6%)
Death†	0	12 (21.0%)

*Samples were collected the same day of organ resection.

†Up to 50 months of follow-up.

CRC, colorectal cancer; HCC, hepatocellular carcinoma.

### Enzyme-linked immunosorbent assay

Serum concentrations of TGFBI were evaluated by ELISA in HD (n=20), and patients with CRC (n=54) and HCC (n=62) using Cusabio kit (Cusabio, Houston, USA) following the manufacturer’s instructions (described in detail in online supplemental material and methods). In addition, secreted TGFBI was determined in supernatants of peripheral CD4 and CD8 T cells, which were purified by negative selection using CD4^+^ T Cell Isolation Kit (Miltenyi Biotec, 130-096-533) and CD8^+^ T Cell Isolation Kit (Miltenyi Biotec, 130-096-495), respectively, following the manufacturer’s instructions.

10.1136/jitc-2025-012668.supp1 Supplementary data

### Survival curve

Levels of TGFBI in the serum of patients with HCC quantified using an ELISA (as described above) were used to generate a Kaplan-Meier survival curve. The curve included high (n=15) and low (n=16) TGFBI groups. The groups were defined based on a cut-off value as the median of serum levels of TGFBI (644.71 ng/mL) in the total HCC patient cohort (n=31), for which survival data were available. The HR and p value were calculated using a log-rank test.

### Tissue microarray assay and immunohistochemistry staining

The panel of tissue microarray (TMA) included healthy tissue controls and biopsies from CRC and HCC of different tumor, node, metastases (TNM) stages (I, II, III, or IV). CRC TMA data includes two cohorts. Clinical data of both cohorts are available at the following links: https://www.tissuearray.com/tissue-arrays/BC051111 (n=90 cases)https://www.tissuearray.com/tissue-arrays/CRC1601csur (n=80 cases). HCC clinical data (reference BC03117) are available at the following link: https://www.tissuearray.com/tissue-arrays/Liver/BC03117 (n=70 cases). Tissue slides obtained from TissueArray.com (Derwood, Maryland, USA) were processed at Research Pathology Platform East (Cancer Research Centre Lyon, Lyon) and stained for TGFBI using an automated immunostainer (Ventana Benchmark Ultra, Roche) (described in detail in online supplemental material and methods).

### Immunofluorescence on CRC and HCC tissues

Formaldehyde-fixed paraffin-embedded tissues from patients with CRC (n=10) and HCC (n=9) were serially sectioned at 2 μm, deparaffinized, and antigen retrieved in Tris/EDTA buffer (pH 9.0). Slides were then blocked with 1% body surface area (BSA) and 5% normal goat serum for 60 min, and double immunofluorescence (IF) staining was performed using anti-TGFBI antibody in combination with anti-epithelial cell adhesion molecule (anti-EpCAM), anti-CD14, anti-CD3, or anti-CD20 antibodies (online supplemental table 1). Confocal microscopy imaging was performed using a Leica TCS-SP8X laser-scanning confocal microscope (Leica Microsystems) equipped with a tunable white light laser source, a 405 nm diode laser, and three photomultiplier tube and two hybrid internal spectral detector channels.^34^ The mean of the double-positive cells detected in five fields for each sample was used in the statistical analysis.

### Isolation of peripheral blood mononuclear cell subsets and TILs

Peripheral blood mononuclear cells (PBMCs) were isolated from HD and patients with CRC and HCC (table 1) as previously described,^35^ and then frozen down in liquid nitrogen. Peripheral CD4^+^ or CD8^+^ T cells were purified by negative selection using CD4^+^ T Cell Isolation Kit (Miltenyi Biotec, 130-096-533) and CD8^+^ T Cell Isolation Kit (Miltenyi Biotec, 130-096-495) following the manufacturer’s instructions, respectively. CD8-depleted PBMCs were obtained by positive subtraction of CD8 with CD8 Microbeads (Miltenyi Biotec, 130-045-201). For the isolation of TILs, surgery-derived TUM and NTUM from CRC and HCC tissues were processed based on previous optimized protocols,^36^ described in the online supplemental material and methods.

### TGFBI neutralization in PBMCs and IHLs ex vivo

Frozen PBMCs of patients with CRC (n=12) and HCC (n=12), and PBMCs from HDs (n=6) used as controls, were quickly thawed in prewarm Roswell Park Memorial Institute (RPMI) 1640 medium. PBMCs were centrifuged at 1200 rpm for 10 min at 4°C and seeded into round bottom 96-well plates (Falcon) at a density of 1–0.5×10^6^ cells per well in a final volume of 200 µL of complete culture medium (RPMI 1640 medium), supplemented with 10% heat-inactivated fetal bovine serum (FBS), L-glutamine (2 mM), penicillin (100 U/mL), streptomycin (100 μg/mL), and sodium pyruvate. PBMCs were seeded alone or with anti-CD3/anti-CD28 beads (Invitrogen) (one bead: five cells), in absence or presence of 3 μg/mL of rabbit (catalog number: 10188-1-AP, Proteintech Europe), or 3 μg/mL of rabbit isotype control IgG (catalog number: NBP2-24893, Novus Biologicals Europe). Similar procedures were performed by using the mouse anti-huTGFBI mAb 18B3 (supplied by AH’s team),^37 38^ previously demonstrated to neutralize TGFBI in vitro and in vivo, and mouse IgG isotype control. Cells were left for 18 hours at 37°C, 5% CO_2_; 4 hours before harvesting cells were incubated with Protein Transport Inhibitor Cocktail (brefeldin A and monensin, eBioscience, Thermo Fisher Scientific) for cytokines detection. At the end of the stimulation, cells were washed by centrifugation at 1200 rpm for 5 min at 4°C in phosphate-buffered saline (PBS, 1×) and stained for FC analysis. IHLs were isolated from NTUM-associated liver margins from patients with primary HCC tumors (n=3) or colorectal tumor liver metastasis (n=3) as described above. IHLs were cultured in complete RPMI 1640 culture medium supplemented with 100 U/mL interleukin (IL)-2 alone or with anti-CD3/CD28 beads (Invitrogen), with or without rabbit anti-huTGFBI (catalog number: 10188-1-AP, Proteintech Europe,) neutralizing antibody at different concentrations (3.0 µg/mL high, 1.0 µg/mL intermediate, and 0.3 µg/mL low) or with rabbit isotype control antibody (rabbit IgG, catalog number: NBP2-24893, Novus Biologicals Europe).

### TGFBI neutralization in CD4^+^ and CD8^+^ isolated T cells

Highly purified CD4^+^ or CD8^+^ T cells (0.1×10^6^/mL) from three HDs were seeded 3-day cultures with anti-CD3/CD28 beads (Invitrogen) at a ratio of 1:8 for all conditions, either alone or in the presence of rabbit anti-huTGFBI (catalog number: 10188-1-AP, Proteintech Europe) at concentrations of 20 µg/mL, 10 µg/mL, and 5 µg/mL, or isotype controls (rabbit IgG, catalog number: NBP2-24893, Novus Biologicals Europe) used at the same concentrations of anti-huTGFBI. Each experiment was performed in triplicate from one HD. At the end of the stimulation, cells were washed by centrifugation at 1200 rpm for 5 min at 4°C in PBS (1×) and stained for FC analysis.

### Human recombinant TGFBI treatment in vitro

The effect of recombinant TGFBI on T cell function, as evaluated in FC analysis, was performed as described in detail in online supplemental material and methods.

### Patient-derived organoids

Patient-derived organoids (PDOs) were established from resected samples from three patients with CRC. Briefly, samples were extensively washed in cold PBS and incubated in Dulbecco’s modified Eagle’s medium (DMEM; Thermo Fisher, Foster City, California, USA) containing 5% penicillin-streptomycin-amphotericin B solution (Thermo Fisher) until processing. Then, tumor tissue was washed in PBS, minced in small pieces (<0.5 mm^3^), and incubated in a digestion medium containing DMEM, 5% penicillin-streptomycin-amphotericin B, and 1.5 mg/mL collagenase type II (Thermo Fisher) at 37°C for 20 min, under shaking. The suspension was filtered with 100 μm Cell Strainer (Falcon, USA), washed twice with PBS, and plated in 60 μL/dome of Matrigel (growth factor-reduced basement membrane extract (BME)) (Corning, New York, USA) in a growth medium described by Sato *et al*^39^ and expanded through passaging every 5–7 days.

### PBMC-PDO co-cultures

PBMCs were isolated from HDs or patients with CRC. For co-culture assays, PBMCs were thawed as previously described.^40^ Briefly, PBMCs were washed and resuspended in T cell thawing medium with 1:1000 benzonase (Sigma-Aldrich), then cultured in T cell culture medium (RPMI 1640, 1% penicillin-streptomycin, 1% glutamine, and 10% human serum) with 150 U/mL of IL-2 overnight. The following day, organoids were treated with 2 mg/mL dispase type II (Sigma-Aldrich), washed, and dissociated with TrypLE Express (Gibco). PBMCs and dissociated organoids (PDO-isolated cells (PDO cells)) were seeded in a 96-well plate at PBMC:PDO cell ratio of 20:1 in organoid medium without nicotinamide supplemented with 10% BME, 5% human serum as previously described.^41^ Where indicated, 10 µg/mL of anti-huTGFBI neutralizing antibody (catalog number: 10188-1-AP, Proteintech Europe) or rabbit isotype control IgG (catalog number: NBP2-24893, Novus Biologicals Europe, Milan, Italy) or 1 µg/mL purified antihuman human leukocyte antigen (HLA)-A, HLA-B, HLA-C antibody (clone W6/32, Biolegend) were added to the culture medium at the specified concentrations. After 72 hours of culture, cell viability/proliferation was determined by evaluating the ATP levels via Cell Titer-Glo Luminescent Cell Viability Assay (Promega, Madison, Wisconsin, USA) with a multimode reader (DTX-880; Beckman Coulter, Brea, California, USA). Culture supernatants were collected and target cell killing was assessed after 72 hours of incubation by quantification of released lactate dehydrogenase (LDH) using an LDH-Glo Cytotoxicity Assay (Promega) according to the manufacturer’s instructions. In CD8-depleted PBMC experiments, cell killing was evaluated by normalizing to LDH baseline values of both effectors (PBMCs) and targets (PDOs) as follows: ((LDH_co-culture_−LDH_PBMC_)/LDH_PDO_). Concentration of TGFBI in culture supernatants was evaluated by ELISA as described above.

PBMCs were isolated from HDs or patients with CRC. For co-culture assays, PBMCs were thawed as previously described.^40^ Briefly, PBMCs were washed and resuspended in T cell thawing medium with 1:1000 benzonase (Sigma-Aldrich), then cultured in T cell culture medium (RPMI 1640, 1% penicillin-streptomycin, 1% glutamine, and 10% human serum) with 150 U/mL of IL-2 overnight. The following day, organoids were treated with 2 mg/mL dispase type II (Sigma-Aldrich), washed, and dissociated with TrypLE Express (Gibco). PBMCs and dissociated organoids (PDO-isolated cells (PDO cells)) were seeded in a 96-well plate at PBMC:PDO cell ratio of 20:1 in organoid medium without nicotinamide supplemented with 10% BME, 5% human serum as previously described.^41^ Where indicated, 10 µg/mL of anti-huTGFBI neutralizing antibody (catalog number: 10188-1-AP, Proteintech Europe) or rabbit isotype control IgG (catalog number: NBP2-24893, Novus Biologicals Europe, Milan, Italy) or 1 µg/mL purified antihuman human leukocyte antigen (HLA)-A, HLA-B, HLA-C antibody (clone W6/32, Biolegend) were added to the culture medium at the specified concentrations. After 72 hours of culture, cell viability/proliferation was determined by evaluating the ATP levels via Cell Titer-Glo Luminescent Cell Viability Assay (Promega, Madison, Wisconsin, USA) with a multimode reader (DTX-880; Beckman Coulter, Brea, California, USA). Culture supernatants were collected and target cell killing was assessed after 72 hours of incubation by quantification of released lactate dehydrogenase (LDH) using an LDH-Glo Cytotoxicity Assay (Promega) according to the manufacturer’s instructions. In CD8-depleted PBMC experiments, cell killing was evaluated by normalizing to LDH baseline values of both effectors (PBMCs) and targets (PDOs) as follows: ((LDH_co-culture_−LDH_PBMC_)/LDH_PDO._. Concentration of TGFBI in culture supernatants was evaluated by ELISA as described above.

### Immunofluorescence on CRC organoids

PDOs were stained with caspase-3/7 activity tracer CellEvent Caspase-3/7 Green Detection Reagent (GFP) (Thermo Fisher Scientific, USA) to detect apoptosis and PBMCs were stained with vital staining CellTracker Red CMPTX Dye (Texas Red) (Invitrogen, USA) and added to cultures. Fluorescence images were acquired with the Axioscope 5 microscope (KMAT, Zeiss) and images were processed with ZEN microscopy software (blue edition). GFP^+^ cells were counted using QuPath software in four fields per sample acquired at 10× magnification. For organoid characterization, PDOs were fixed with 4% paraformaldehyde, permeabilized with 0.5% Triton X-100 in PBS for 1 hour at room temperature (RT), and blocked with 3% BSA, 3% FBS, and 0.5% Triton X-100 in PBS for 1 hour at RT. PDOs were then stained for CDX2, CK20, EpCAM, and mucin 1 antibodies (online supplemental table 1) overnight at 4°C. The following day, PDOs were incubated with goat antirabbit IgG Alexa Fluor 488, goat antimouse IgG Alexa Fluor 555, and Hoechst (Invitrogen) overnight at 4°C. Slides were permanently mounted with Prolong-Gold Antifade (Thermo Fisher) and analyzed with Zeiss LSM900 confocal microscope.

### Flow cytometry analysis

Before surface staining, PBMCs or isolated (CD4^+^ or CD8^+^) T cells were incubated with Fixable Viability Dye eFluor780 (eBiosciences) for 30 min at RT to exclude dead cells from the analysis. After washing, cells were incubated with several labeled mAbs specific to surface markers listed in online supplemental table 2 for 20 min at 4°C in PBS containing 2% FBS. To analyze cytokine production, cells were fixed and permeabilized using the BD Cytofix/Cytoperm Fixation/Permeabilization Solution Kit (BD Biosciences) at 4°C for 20 min, washed, and stained with mAbs to TGFBI, interferon (IFN)-γ, and tumor necrosis factor (TNF)-α (online supplemental table 2) for 20 min at 4°C in BD Perm/Wash buffer (BD Biosciences). Cells were washed and acquired with LSRFortessa cytometer (BD Biosciences). Cells isolated from liver resections and stimulated as described above were incubated with brefeldin A and monensin for 16 hours at 37°C, 5% CO_2_. Cells were then harvested, stained with fixable blue live/dead reagent (Thermo Fisher, catalog number L23105), followed by mAbs as listed in online supplemental table 2 and analyzed by FC using a Cytek Aurora spectral cytometer (Cytek). All samples were analyzed with FlowJo software V.10.0.8r1 (TreeStar). Gating strategies are illustrated in online supplemental figure 5 and figure 11A.

## Statistical tests

For ELISA, an unpaired two-tailed Student’s t-test was performed for the HD and CRC/HCC groups. The probability of survival was calculated using the log-rank test, considering the median of TGFBI serum levels evaluated by ELISA as cut-off. Quantification of TGFBI expression on TMA across different cancer stages was performed using a one-way analysis of variance (ANOVA) using Tukey’s multiple comparison tests. TGFBI quantification by IF staining was calculated using a paired two-tailed Student’s t-test. For the frequency and mean fluorescence intensity (MFI) of TGFBI-expressing immune cells were analyzed using one-way ANOVA with Tukey’s multiple-comparison tests, and paired two-tailed Student’s t-test was used to compare patients’ NTUM and TUM cell frequencies. For in vitro neutralization assays, a paired two-tailed Student’s t-test and one-way ANOVA were performed according to the experimental scheme. In experiments using TGFBI recombinant protein, both ex vivo and in vitro, an ordinary one-way ANOVA test using Tukey’s multiple comparison tests was used. The same test was also used in organoid experiments.

## Results

### TGFBI is expressed in tumor tissues, and its serum levels are associated with poor prognosis

**Figure 2:**
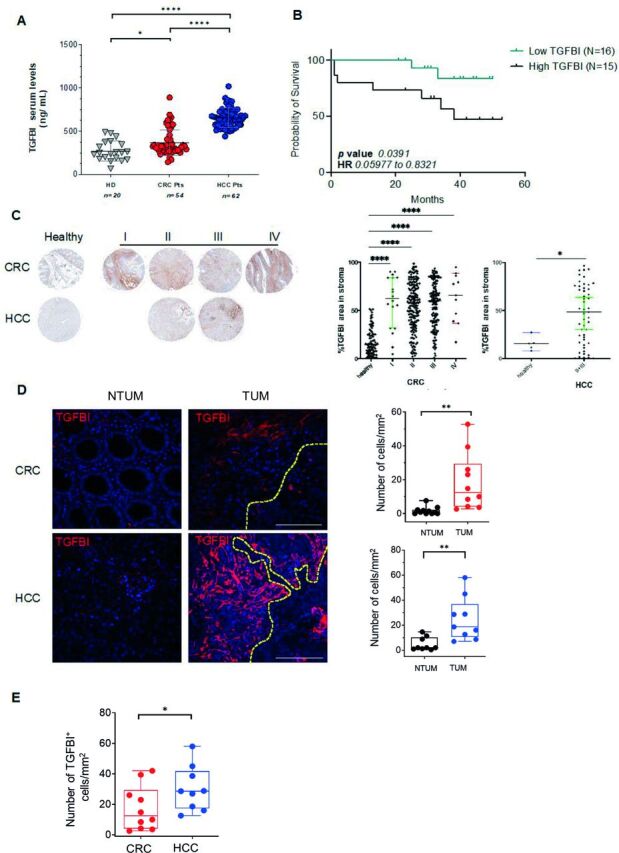
TGFBI expression is higher in serum and TUM tissues and is associated with poor survival. (A) Serum levels of TGFBI (ng/mL) detected by ELISA, in HDs (n=20), and patients with CRC (n=54) and HCC (n=62). Statistical significance was calculated by ordinary one-way ANOVA test. (B) Kaplan-Meier survival curves representing high (n=15) and low (n=16) groups based on serum TGFBI levels by ELISA quantification (cut-off as median 644.71 ng/mL) in patients with HCC (n=31). HR (0.2230) and p value (0.0391) were calculated by log-rank test. (C) Representative of TGFBI staining in TMA. Quantification of TGFBI percentage in CRC and HCC. For the CRC cohort, a total of 498 samples were analyzed including 110 samples from healthy tissues. Most of the sections available corresponded to stage II (n=183) and stage III (n=143) carcinoma. 18 valid samples were analyzed for stage I and 11 stage IV biopsies. For the HCC cohort, a total of 56 tissues were analyzed, including five samples from healthy tissues. Pooled sections corresponding to stage II (n=19) and stage III (n=32) carcinoma were represented. One-way ANOVA and t-test were performed. (D) Representative IF images for TGFBI-expressing cells (red) in NTUM and TUM tissue of patients with CRC or HCC are shown (original magnification 40×, scale bar 30 μm). Nuclei are counterstained with Hoechst (blue). Dotted lines divide stromal zones (above) from non-stromal zones (below). On the right, quantitative analysis of TGFBI-expressing cells from 10 independent patients with CRC and nine HCC is shown. (E) Quantitative analysis of TGFBI-expressing cells in HCC and CRC TUM tissues. For D and E, levels of significance were determined by paired and unpaired two-tailed Student’s t-test, respectively. *P<0.05, **p<0.01, ****p<0.0001. ANOVA, analysis of variance; CRC, colorectal cancer; HCC, hepatocellular carcinoma; HD, healthy donor; IF, immunofluorescence; Pts, patients; NTUM, non-tumor tissue; TGFBI, transforming growth factor β induced; TMA, tissue microarray; TUM, tumor tissue.

Serum TGFBI levels, as measured by ELISA, were significantly higher in patients with CRC and HCC^8 9^ (table 1) than in HDs, with highest levels in patients with HCC (figure 2A). Notably, the serum levels of TGFBI were associated with poor survival in a smaller cohort of patients with HCC (n=31), for which the survival data along a 50-month follow-up after surgery were available (figure 2B). Survival analysis on the CRC cohort was not eligible since all patients were alive during our follow-up after surgery.^42^ Taken together, these data support that serum TGFBI can represent a valid prognostic biomarker of fatal cancer evolution.^22 25 43^ These data were further sustained in situ using TMA analysis in a larger cohort of patients with CRC and HCC. The panel of TMA included healthy tissue as controls and biopsies from CRC and HCC of different TNM stages (I, II, III, or IV). Stromal TGFBI expression was significantly higher in all CRC (stages I–IV) and HCC (stages II–III) compared with healthy tissues (figure 2C), although no significant differences were observed among the CRC and HCC stages.

In line with data above, in situ IF analysis showed significantly higher TGFBI expression in TUM compared with NTUM tissues from patients with CRC and HCC and higher expression in HCC compared with CRC (figure 2D, E). A substantial proportion of EpCAM-positive tumor cells expressed TGFBI in both CRC and HCC (online supplemental figure 1A-D), thus indicating a contribution of tumor cells to TGFBI production.

### TGFBI is expressed by PBMCs and multiple TUM-infiltrating or NTUM-infiltrating immune cell subsets in patients with CRC and HCC

**Figure 3:**
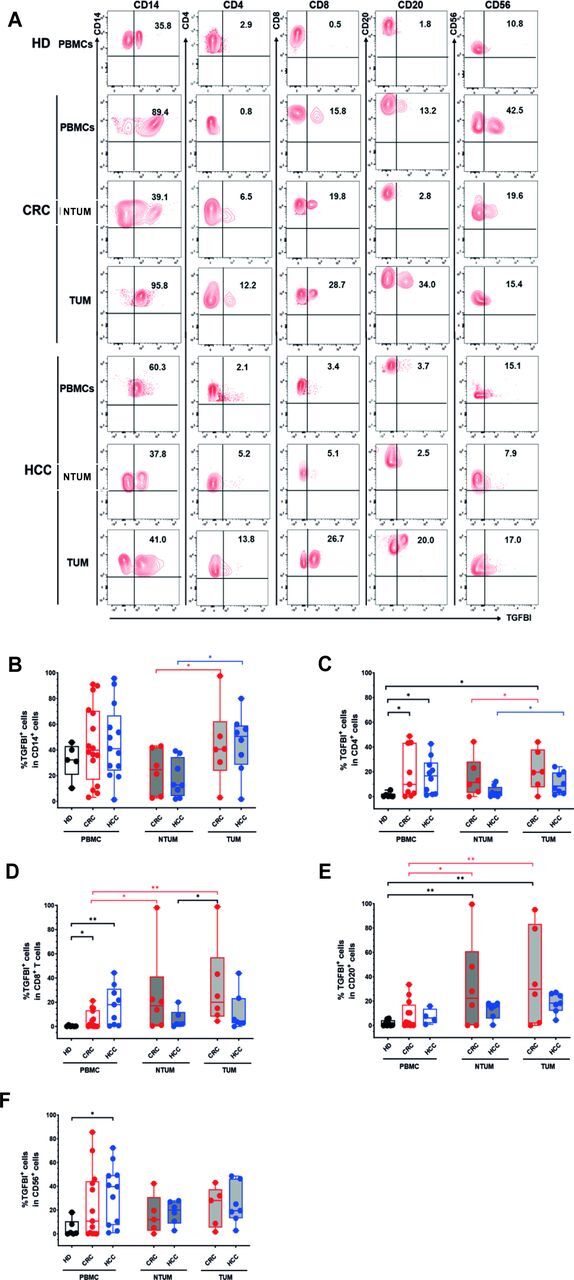
TGFBI is expressed by peripheral, TUM-infiltrating, or NTUM-infiltrating immune cell populations from patients with tumor. (A) Representative flow cytometry analysis of TGFBI expression as percentage of CD14^+^, CD4^+^, CD8^+^, CD20^+^, CD56^+^ cells within PBMCs, and within NTUM-infiltrating or TUM-infiltrating MCs from patients with CRC (PBMC n=12, NTUM n=6, TUM n=6) (top) or HCC (PBMC n=12, NTUM n=8, TUM n=8) (bottom). (B–F) Box plots indicating as median maximum and minimum frequencies of TGFBI^+^ cells in CD14^+^ (B), CD4^+^ (C), CD8^+^ (D), CD20^+^ (E), or CD56^+^ (F) cells within PBMCs of HDs, or PBMCs, NTUM-infiltrating or TUM-infiltrating MCs from patients with CRC or HCC. Differences between HD-PBMCs and patients’ PBMCs, NTUM-immune or TUM-immune MCs were calculated by ordinary one-way analysis of variance Tukey’s multiple comparison tests (indicated in black) and differences within patient’s NTUM versus PBMCs, TUM versus PBMCs, and TUM versus NTUM by paired two-tailed Student’s t-test (indicated in red for CRC and in blue for HCC). *P<0.05, **p<0.01. CRC, colorectal cancer; HCC, hepatocellular carcinoma; HD, healthy donor; MC, mononuclear cell; NTUM, non-tumor tissue; PBMCs, peripheral blood mononuclear cells; TGFBI, transforming growth factor β induced; TUM, tumor tissue.

IF analysis showed a consistent number of TGFBI^+^CD14^+^ monocytes that were significantly higher in TUM than in NTUM from patients with HCC and close to significantly in patients with CRC (online supplemental figure 2A-D). Importantly, TGFBI^+^CD3^+^ T cells were significantly higher in TUM than in NTUM from patients with CRC and HCC, whereas TGFBI^+^CD20^+^ B cells tended to increase without reaching statistical difference (online supplemental figures 3A-D and 4A-D). FC analyses (gating strategy in online supplemental figure 5A, B) showed that TGFBI was expressed by notable proportions of CD14^+^ monocytes, CD4^+^ T cells, CD8^+^ T cells, CD20^+^ B cells, and CD56^+^ NK cells (figure 3A-F, online supplemental figure 6A-E). It was mainly expressed by CD14^+^ monocytes, with significant differences between the TUM-derived and the NTUM-derived cells, but without significant difference with their peripheral counterparts, in patients with HCC and CRC (figure 3B). However, we observed significant differences for the following comparisons: TGFBI^+^CD4^+^ T cells (CRC or HCC PBMCs vs HD PBMCs; CRC TUM vs HD PBMCs; CRC TUM vs CRC NTUM; HCC TUM vs HCC NTUM) (figure 3C), TGFBI^+^CD8^+^ T cells (CRC or HCC PBMCs vs HD PBMCs; CRC TUM vs CRC PBMCs; CRC NTUM vs CRC PBMCs) (figure 3D), TGFBI^+^CD20^+^ B cells (CRC NTUM or TUM vs HD PBMCs; CRC NTUM or TUM vs CRC PBMCs) (figure 3E), TGFBI^+^CD56^+^ cells (HCC PBMCs vs HD PBMCs). Notably, a significant difference was observed between cells derived from TUM and NTUM in TGFBI expression intensity (MFI) (online supplemental figure 6A-F). In addition, we provided evidence that TGFBI was detected in the supernatants of highly purified CD4^+^ or CD8^+^ T cells, confirming that it was secreted by T cells ex vivo (online supplemental figure 7).

### TGFBI inhibits T cell responses and its neutralization restored them

**Figure 4:**
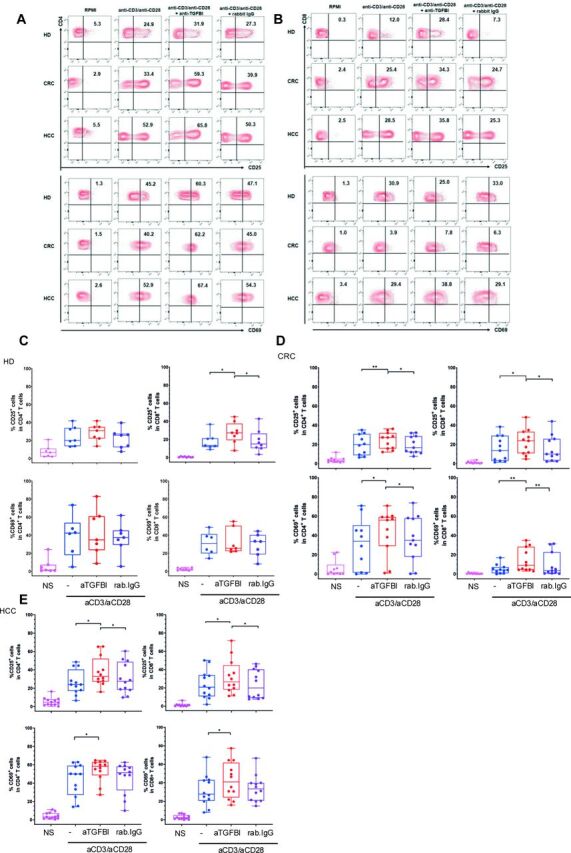
Neutralizing anti-TGFBI antibody increases activation of CD4^+^ or CD8^+^ T cells in PBMCs from HDs, and patients with CRC and HCC. (A, B) Representative flow cytometry contour plots of CD25 and CD69 of non-stimulated or anti-CD3/CD28-stimulated CD4^+^ (A) and CD8^+^ (B) T cells (0.1×10^6^ cells/well) within PBMCs of HDs (left), and patients with CRC (right) and HCC (bottom), in the presence or absence of the neutralizing anti-TGFBI antibody or the corresponding isotype control (rabbit IgG). (C–E) Box plots indicating median maximum and minimum frequencies of CD25^+^ or CD69^+^ cells gated in CD4^+^ or CD8^+^ T cells in PBMCs from HDs (n=7) (C), and patients with CRC (n=10) (D) and HCC (n=12) (E), in the presence or absence of the neutralizing anti-TGFBI antibody or the corresponding rabbit isotype control. *P<0.05, **p<0.01 by paired two-tailed Student’s t-test. CRC, colorectal cancer; HCC, hepatocellular carcinoma; HD, healthy donor; NTUM, non-tumor tissue; PBMC, peripheral blood mononuclear cell; TGFBI, transforming growth factor β induced; TUM, tumor tissue.

**Figure 5:**
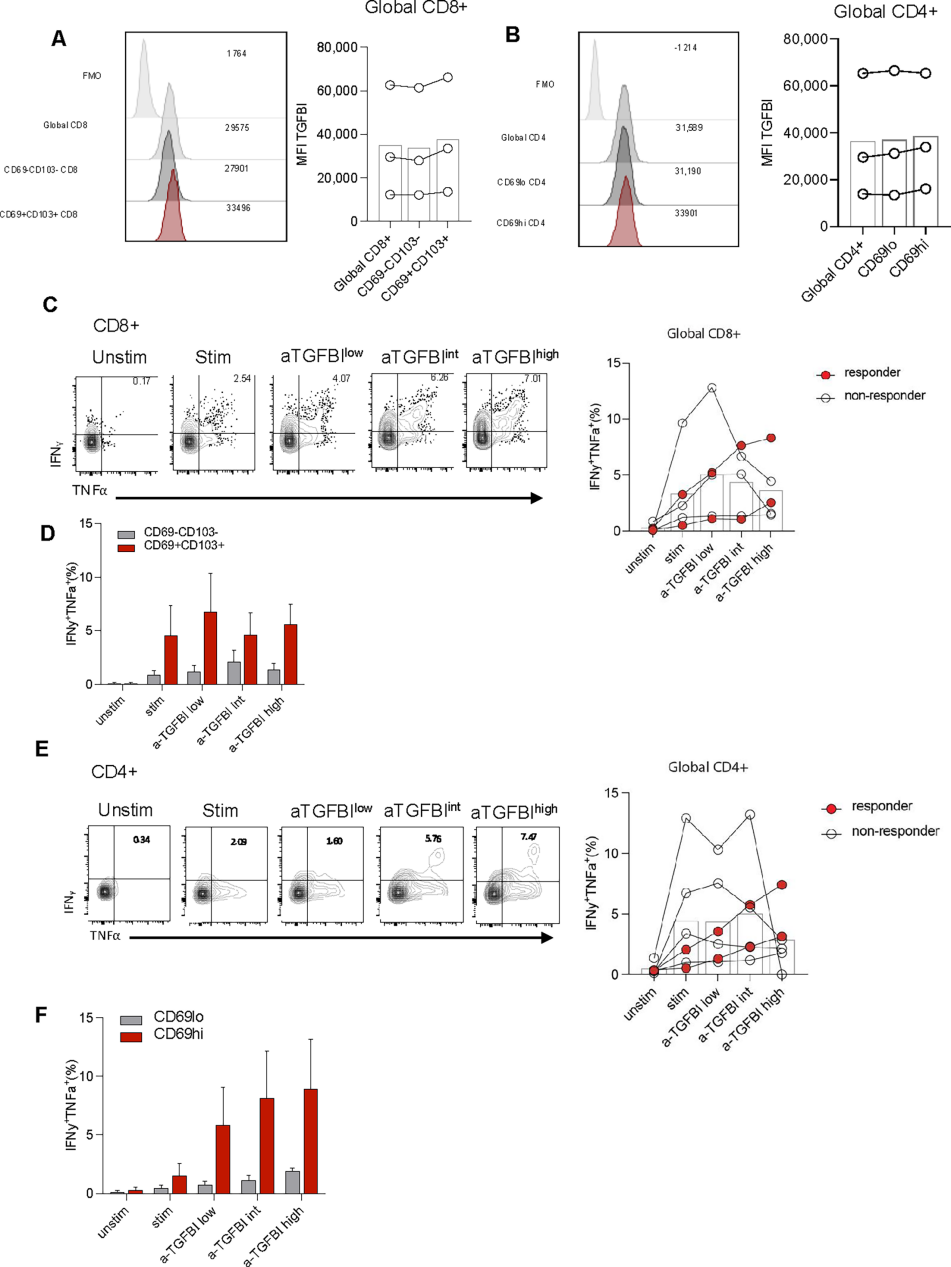
Expression of TGBI in intrahepatic T cells and their functional response to polyclonal stimulation on TGFBI neutralization. (A–B) Ex vivo FC analysis of TGFBI expression in T cells isolated from tumor-free liver margins resected from patients with HCC (n=3) or CRC liver metastasis (n=3). Histograms show TGFBI expression (MFI) on global liver CD8^+^ T cells, liver-infiltrating CD69^–^CD103^–^CD8^+^ T cells, and liver-resident CD69^+^CD103^+^CD8^+^ T cells compared with FMO control (A) or on global liver CD4^+^ T cells, liver-infiltrating CD69^lo^ CD4^+^ T cells and liver-resident CD69^hi^ CD4^+^ T cells (B) and summary plots of n=3 (right). (C–F) Intrahepatic lymphocytes isolated from non-tumor-associated liver tissue were unstimulated or anti-CD3/CD28-stimulated in the presence or absence of the neutralizing anti-TGFBI antibody at low (0.3 µg/mL), intermediate (1.0 µg/mL), and high (3.0 µg/mL) concentrations, overnight and analyzed by FC. (C and E) Representative plots of IFN-γ and TNF-α co-expression in global CD8^+^ T cells (C) and global CD4^+^ T cells (E) and summary graph for full cohort (n=5, red circles highlight donors with dose-dependent responses to TGBI blockade). (D and F) Summary bar graph showing frequency of IFN-γ^+^TNF-α^+^ liver-infiltrating and tissue-resident CD8^+^ T cell (D) and CD4^+^ T cell (F) fractions on polyclonal stimulation±TGBI blockade (n=4). CRC, colorectal cancer; FC, flow cytometry; FMO, fluorescence minus one; HCC, hepatocellular carcinoma; IFN, interferon; MFI, mean fluorescence intensity; Stim, stimulated; TNF, tumor necrosis factor; TGFBI, transforming growth factor β induced; Unstim, unstimulated.

A recombinant form of TGFBI (rTGFBI) significantly inhibited CD4^+^ or CD8^+^ T cell activation and differentiation in PBMCs from HDs in vitro. rTGFBI induced decreased T-bet (the key transcription factor for the IFN-γ production),^44^ IFN-γ and granzyme B expression (evaluated as both percentage and MFI by FC) following 20-hour stimulation with suboptimal concentrations of anti-CD3 and anti-CD28 mAbs (polyclonal TCR-dependent stimulation) (online supplemental figure 8A-E). These data support that exogenous TGFBI can alter CD4 and CD8 T effector cell differentiation and function.^28^ Then, based on the evidence showing that TGFBI is expressed by tumor-infiltrating immune cells (figure 3), we questioned whether the blocking of TGFBI may modify T cell activation. To test this hypothesis, we performed a series of experiments ex vivo, in which PBMCs from patients with CRC or HCC (expressing TGFBI at the level of monocytes, T cells, B cells, and NK cells) (figure 4) were exposed to polyclonal TCR-dependent stimulation for 20 hours, in presence or absence of different concentrations of a neutralizing anti-TGFBI antibody or the corresponding isotype. Strikingly, the addition of the neutralizing anti-TGFBI antibody alone (rabbit anti-huTGFBI (Proteintech Europe)) (but not the corresponding isotype) significantly increased the activation of both CD4^+^ and CD8^+^ T cells in PBMCs derived from patients with cancer, in terms of increased expression of CD25 and CD69 as activation markers (figure 4, online supplemental figure 9A-C). Similarly, both CD4^+^ and CD8^+^ T cell activation was validated by using a further neutralizing anti-TGFBI mAb (18B3)^38^ (online supplemental figure 10). Next, we confirmed that TGFBI was expressed in both CD8^+^ and CD4^+^ T cells isolated from human liver samples (figure 5A, B). Interestingly, both tissue-resident memory (TRM) T cells (CD8^+^CD69^+^CD103^+^ or CD4^+^CD69^hi^ T cells (figure 5A,B; gating strategy in online supplemental figure 11A)^41–43^ and their recirculating CD103^–^ or CD69^–^ infiltrating counterparts expressed TGFBI at similar levels (figure 5A, B). On TGFBI neutralization, two of five enrolled patients showed a clear, dose-dependent increase in frequency of IFN-γ and TNF-α production on global CD8^+^ and CD4^+^ T cells (figure 5C, E). In these two patients, we isolated sufficient liver T cells to also show an enhanced dose-dependent response to TGFBI neutralization compared with matched concentrations of an isotype control blocking antibody (representative example, online supplemental figure 11B, C). Both CD8^+^ and CD4^+^ TRM cells showed an enhanced response to the baseline mitogen stimulation compared with their infiltrating non-TRM (recirculating) counterparts, as previously reported by our group and others.^45 46^ In those samples, where sufficient cells were available to examine the impact of TGFBI neutralization within resident and infiltrating intrahepatic T cell subsets (n=4) (figure 5D,F), we observed a trend toward a more marked increase of IFN-γ^+^/TNF-α^+^ cells in the CD69^hi^CD4^+^ T cell resident subset, as compared with the CD69^lo^ ones (figure 5F), although the limited sample size did not allow statistical analysis. These data suggest that TGFBI neutralization potentially improves intrahepatic CD69^hi^CD4^+^ T cell effector functions by producing high levels of inflammatory cytokines. The lack of T cell response at higher anti-TGFBI antibody concentration in some patients is likely due to saturation by lower antibody concentration masking dose-dependent effect.

### TGFBI-expressing T cells counterinhibit their own differentiation and effector functions

**Figure 6:**
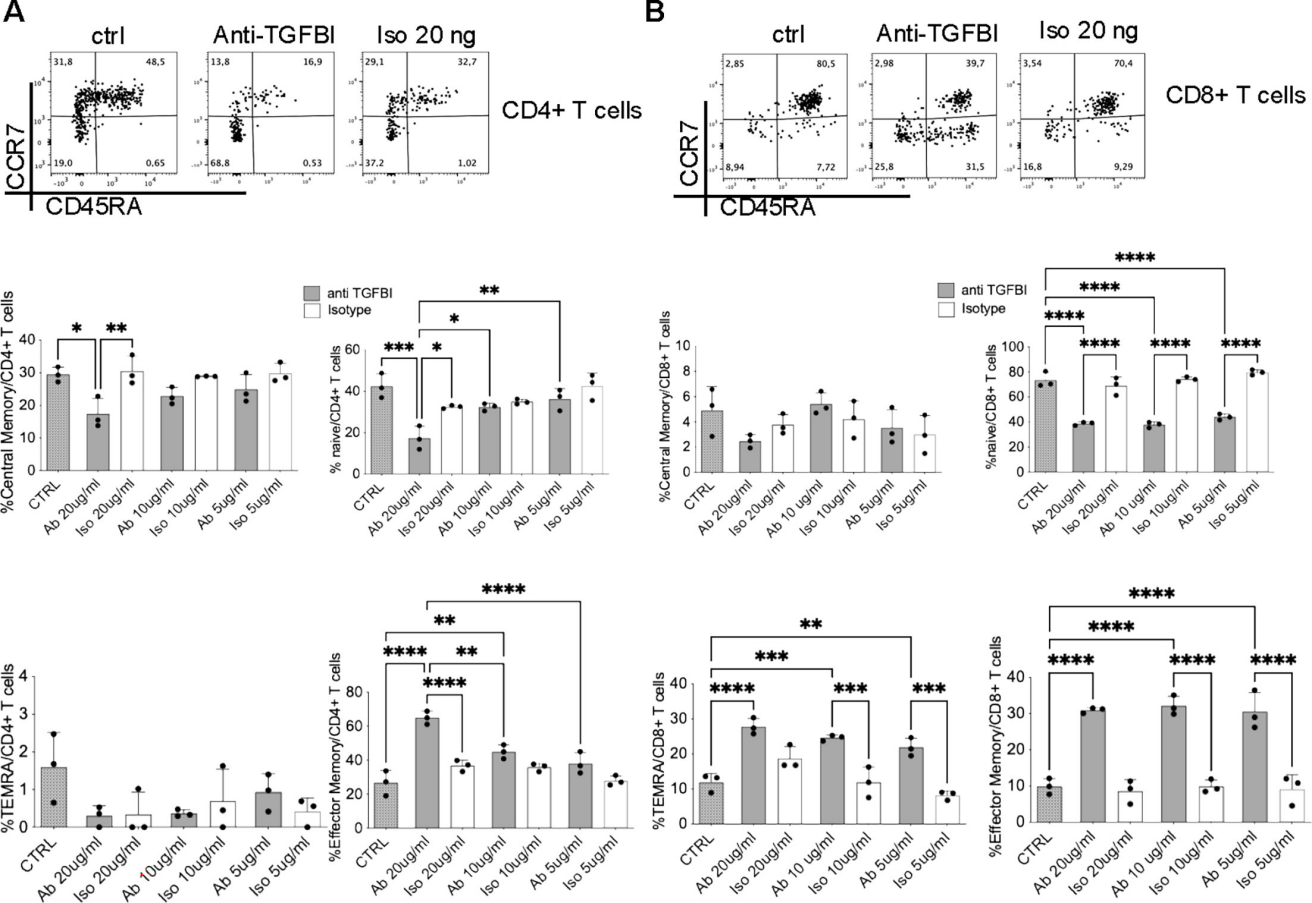
Neutralization of TGFBI unleashes differentiation and effector functions of freshly isolated CD4^+^ or CD8^+^ T cells. (A, B) Representative flow cytometry dot plot analysis of CCR7^+^ and CD45RA^+^ cells within freshly isolated CD4^+^ or CD8^+^ T cells from HDs, which had been non-stimulated (CTRL) or anti-CD3/CD28-stimulated in the presence of the neutralizing anti-TGFBI antibody (anti-TGFBI) or the corresponding Iso. Cells (0.1×10^6^ cells/well), gated in freshly isolated CD4^+^ (A) or CD8^+^ (B) T cells from one representative HD are shown (upper panels). Results of naïve (CCR7^+^CD45RA^+^), central memory (CCR7^+^CD45RA^–^), EM (CCR7^–^CD45RA^–^), EMRA (CCR7^–^CD45RA^+^) within freshly isolated CD4^+^ or CD8^+^ T cells from three independent HDs are shown. *P<0.05, **p<0.01, ***p<0.001, ****p<0.0001 ordinary one-way analysis of variance test using Tukey’s multiple comparison tests. CCR7, C-C chemokine receptor type 7; CTRL, control; CRC, colorectal cancer; EM, effector memory; EMRA, effector memory RA; HD, healthy donor; Iso, isotype control; TGFBI, transforming growth factor β induced.

To support the possibility that TGFBI-producing T cells can counter-regulate themselves, we performed neutralizing experiments in vitro, with freshly purified CD4^+^ or CD8^+^ T cells expressing TGFBI from HDs that were exposed or not to the polyclonal TCR-dependent stimulation for 3 days, in the presence or absence of different concentrations of neutralizing anti-TGFBI antibody or the corresponding isotype control. Under these conditions, the addition of different concentrations of neutralizing anti-TGFBI antibody increased the differentiation and function of T cells in vitro compared with the isotype control (figure 6). Indeed, we found a significant decrease in the percentage of naïve (CCR7^+^CD45RA^+^) and central memory (CM; CCR7^+^CD45RA^–^) and a parallel significant increase in the percentage of effector memory CD45RA^+^ (EMRA; CCR7^–^CD45RA^+^) CD4^+^ T cells (figure 6A). No statistical differences were observed in effector memory (EM; CCR7^–^CD45RA^–^) CD4^+^ T cell compartment. Of note, a significant decrease in the percentage of naïve (CCR7^+^CD45RA^+^) cells, associated with a significant increase in both EM (CCR7^–^CD45RA^–^) and EMRA (CCR7^–^CD45RA^+^) CD8^+^ T cells, was observed in the presence of anti-TGFBI (figure 6B). No statistical differences were instead observed in CM (CCR7^+^CD45RA^–^) CD8^+^ T cell population.

### TGFBI neutralization improves T cell killing of CRC patient-derived organoids

**Figure 7:**
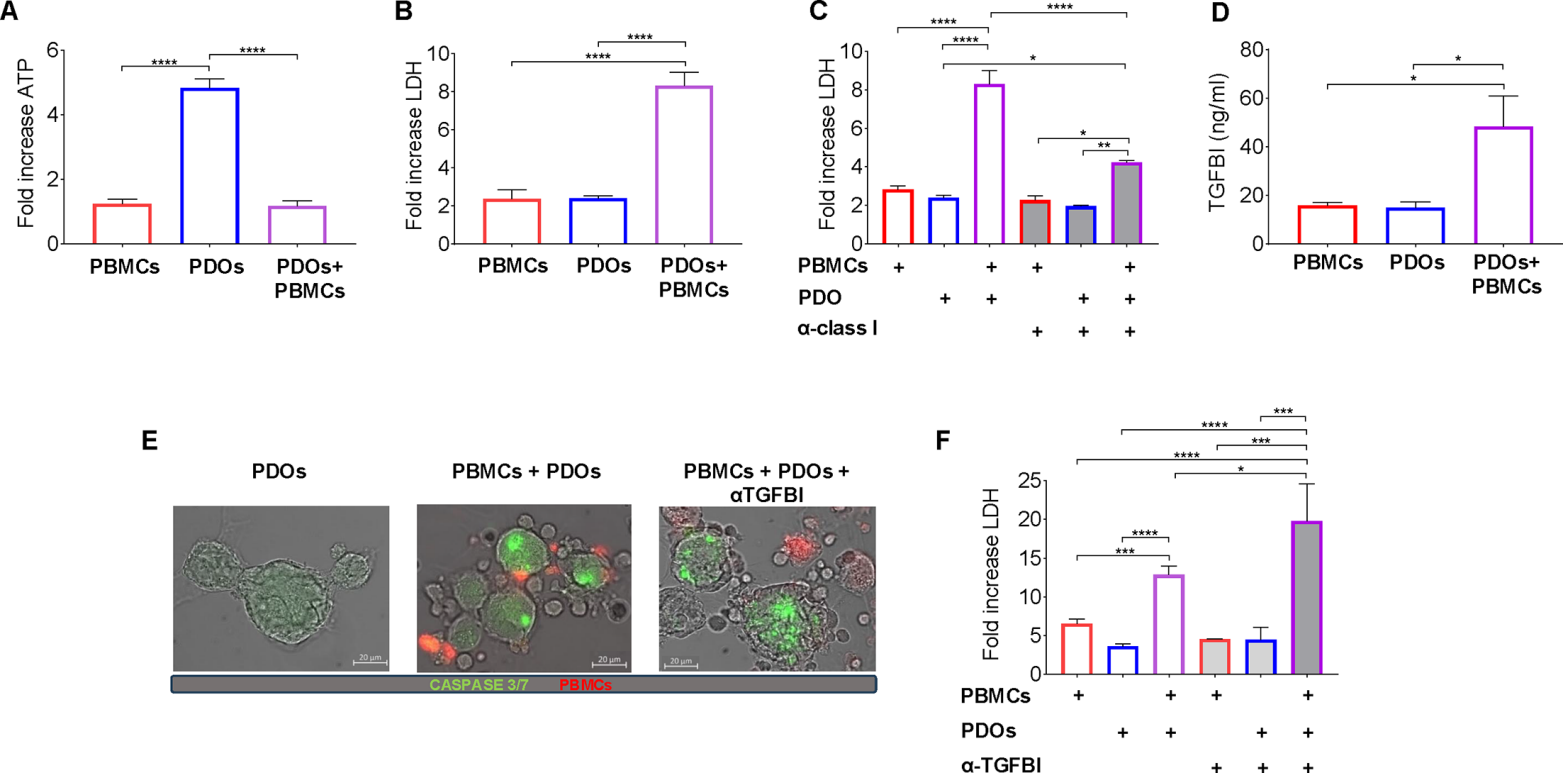
TGFBI neutralization improves T cell killing of CRC patient-derived organoids. (A) Cell viability of PBMC and PDO cell co-cultures. Matched PBMCs (red bar) and PDO cells (PDOs) (blue bar) derived from three patients with CRC were cultured alone or co-cultured (violet bar) at an effector-to-target (E:T) ratio of 20:1. After 72 hours, cell viability was assessed by ATP quantification (Cell Titer Glo assay). Histograms represent the fold increase at 72 hours, normalized to day 0. Data represent the mean±SEM of three independent experiments. (B) Cytotoxicity was measured after 72 hours of co-culture by quantifying LDH release. Data represent the mean±SEM of three independent experiments. (C) Matched PBMCs (red bar) and PDOs (blue bar) were cultured alone or co-cultured (violet bar) at an E:T ratio of 20:1, in the presence (gray-filled bars) or absence (empty bars) of an anti-MHC class I antibody (1 μg/mL). LDH release was measured after 72 hours to assess target-cell killing. (D) TGFBI concentration (ng/mL) in culture supernatants was measured using ELISA in matched PBMCs and PDOs alone and co-cultured, as described in (A). Data represent the mean±SEM of three independent experiments. (E) Representative images of PBMC/PDO co-cultures with or without the neutralizing anti-TGFBI antibody (10 µg/mL). PBMCs were stained with CMPTX Dye (red) and PDOs were labeled with caspase-3/7 (green), (bars 20 μm). (F) LHD assay of PBMCs (n=5) (red bar) and PDOs (blue bar), cultured alone or in co-culture (violet bar) at an E:T ratio of 20:1, in the presence (gray-filled bars) or absence (empty bars) of anti-TGFBI antibody (10 µg/ml). Data represent the mean±SEM of five independent experiments with PBMCs. *P<0.05, **p<0.01, ***p<0.001, ****p<0.0001 by ordinary one-way analysis of variance Tukey’s multiple comparison tests. CRC, colorectal cancer; E:T, effector-to-target; LDH, lactate dehydrogenase; MHC, major histocompatibility complex; PBMC, peripheral blood mononuclear cell; PDO, patient-derived organoid; TGFBI, transforming growth factor β induced.

CRC PDOs were isolated from surgical samples and characterized (online supplemental figure 12), as previously described.^39^ In a three-dimensional (3D) co-culture model, PDO cells from three independent patients with CRC were seeded along with autologous PBMCs to explore the possibility that cytotoxic T cells can recognize and kill cancer cells. After 72 hours, cell viability was evaluated by measuring the intracellular levels of ATP (Cell Titer-Glo assay—Promega), while the levels of released LDH in the supernatants were measured to assess cytotoxicity. We observed a reduced viability in PDO cell/PBMC co-cultures compared with PDO cell monocultures, paralleled by a significant increase of LDH levels (figure 7A, B). To confirm that, the observed killing was mediated by cytotoxic T cells (CTLs) targeting PDO cells, blocking experiments were conducted using an antibody recognizing non-polymorphic region of major histocompatibility complex (MHC) class I molecules. Notably, the addition of the blocking MHC class I antibody (but not of the corresponding isotype control) to PDO cell/PBMC co-cultures, but not to PDO cell or PBMC monocultures, significantly reduced LDH levels (figure 7C, online supplemental figure 13). This finding indicates that PDO cell killing was mainly driven by CTLs through the recognition of agnostic antigenic epitope/class I molecule complexes on cancer cells. To further confirm the role of CTLs, we performed co-culture experiments with CD8-depleted PBMCs. LDH release values in CD8-depleted co-cultures were comparable to baseline PDO levels (ratio ~1), confirming that the killing effect is mainly mediated by CD8^+^ CTLs (online supplemental figure 14). We next explored the role of TGFBI in PDO recognition and killing by CTLs. The levels of secreted TGFBI in the PDO cell/PBMC co-cultures were significantly higher compared with monocultures (figure 7D). To monitor PDOs and PBMCs interaction and tumor cells’ apoptosis, PBMCs were labeled with the CMPTX Dye (red), while PDO cells were stained with caspase-3/7 dye that provides a green fluorescent signal on specific activation of caspase-3/7. IF imaging confirmed increased tumor cell killing in PDO cell/PBMC co-cultures and on anti-TGFBI blockade (figure 7E, online supplemental figure 15). This observation was highlighted by the evidence that neutralization of secreted TGFBI in the PDO cell/PBMC co-cultures significantly improved cancer cell killing, as shown by the increased levels of LDH release (figure 7F, online supplemental figure 13). Altogether, these data suggest that CTLs kill cancer cells, as indirectly highlighted by caspase activation in cancer cells, and that TGFBI neutralization can enhance the killing function.

## Discussion

TGFBI is an ECM protein that is oversecreted in the TME of several solid tumors by its N-terminal secretory signal (aa 1–23).^17 47^ It promotes tumor cell survival, angiogenesis, metastasis, and immunosuppression through the interaction with various integrins.^20–23 28–31^ Previous studies demonstrated that CAFs and macrophages produce TGFBI suppressing T cell responses, which were restored by TGFBI neutralization in cancer mouse models.^28–31^ However, the extent to which TGFBI interferes with T cell function and differentiation, and whether tumor-associated T cells themselves contribute to TGFBI production—thereby counter-regulating their own responses in a manner reversible by TGFBI blockade in humans—remains unclear.

Here, we provide novel evidence highlighting a key role of elevated TFGBI levels in cancer progression. Notably, we demonstrate that TGFBI sources are tumor cells and tumor-associated monocytes, as previously reported,^29 31 48^ and various adaptive and innate immune cells, including peripheral as well as TUM-infiltrating and NTUM-infiltrating CD4^+^ T cells, CD8^+^ T cells, B cells, or NK cells from patients with cancer. Importantly, TGFBI can act as a sIC that counterinhibits tumor-associated T cells. This potential sIC role was mechanistically supported by ex vivo functional analyses showing that the addition of neutralizing anti-TGFBI antibodies enhanced T cell activation (following polyclonal TCR stimulation) in CRC-derived or HCC-derived PBMCs containing TGFBI-expressing monocytes, CD4^+^ T cells, CD8^+^ T cells, B cells, and NK cells. Furthermore, TGFBI was also expressed by liver-resident CD8^+^ and CD4^+^ T cell subpopulations (including the TRM) from patients, and its neutralization enhanced their own functions directly ex vivo.

Consistent with an autoregulatory TGFBI loop in T cells, TGFBI neutralization enhanced functions by freshly isolated TGFBI-expressing CD4^+^ or CD8^+^ T cells (ie, deprived from other cellular sources of TGFBI) following TCR stimulation. Notably, TGFBI blockade promoted their differentiation from naïve (CCR7^+^CD45RA^+^) or CM (CCR7^+^CD45RA^–^) to EM (CCR7^–^CD45RA^–^) or EMRA (CCR7^–^CD45RA^+^) T cells. Given that CCR7 is a key lymphoid homing receptor favoring T cell recirculation via lymphoid organs and restricting T cell migration into peripheral inflamed tissues,^49 50^ these findings suggest that TGFBI may limit T cell migration into the tumor site, thereby contributing to the immune-excluded (cold) TME. This aligns with our previous data suggesting that the TME impairs terminal differentiation of T effector cells in various human tumors.^35 45^

Moreover, the improvement of cancer cell killing by MHC class I-restricted cytotoxic T lymphocytes in 3D CRC organoids following TGFBI neutralization supports the role of TGFBI as a sIC and highlights the potential of TGFBI blockade to restore antitumor immunity by CTLs in humans. PDOs recapitulate self-organizing tumor architecture and enable study of functional interactions with autologous antitumor immune responses.^40 41^ Thus, PDOs provide a powerful platform for screening multiple pharmacological agents, including neutralizing antibodies against TGFBI or other potential sICs identified in TME secretome by mass spectrometry-based analysis (manuscript in preparation), paving the way for novel precision or even personalized immunotherapies. Collectively, our findings provide a mechanistic basis for previous observations in mouse models, where TGFBI neutralization increased activated TILs and reduced tumor burden.^28–31^

Given TGFBI expression by a broad range of both TUM-infiltrating and NTUM-infiltrating immune cell populations, it likely acts as a sIC establishing a generalized immunosuppressive milieu throughout the TUM and adjacent NTUM. Both CRC and HCC NTUM districts commonly exhibit chronic low-level inflammation, a known driver of tumor development,^51 52^ suggesting that TGFBI plays a key role in linking chronic inflammation and tumor development. Additionally, the detection of TGFBI^+^ immune cells in PB of patients with cancer implies that they may recirculate systemically, potentially contributing to systemic immunosuppression via TGFBI secretion.

Whether TCR activation directly induces TGFBI transcription in T cells or acts indirectly by promoting cytokines such as TGF-β, critical for TGFBI production,^17 46 52^ remains an intriguing area for future research. Importantly, secreted TGFBI triggers an inhibitory signaling loop through binding to T cell surface integrins, ultimately leading to phosphorylation of the inhibitory tyrosine Y505 on the TCR-associated kinase Lck, thereby suppressing T cell activation in vitro and in vivo.^28 29^

In conclusion, we propose that TGFBI produced by activated T cells can inhibit their own activities in an autocrine manner and support paracrine immunosuppression via secretion from neighboring cells in inflamed and tumor tissues. Our data suggest that secreted TGFBI hinders differentiation into migratory effector (CCR7^–^CD45RA^–^ or CCR7^–^CD45RA^+^) T cells, likely contributing, along with its capacity to interfere with various integrins,^20–23 28–31^ to limiting T cell migration into the tumor site, and to establishing an immune-excluded (cold) TME. Consistently, our results provide a mechanistic basis for the spatial multi-omics study in human CRC specimens, which identified TGFBI as a relevant component of an immune exclusion signature linked to T cell exhaustion and poor patient outcomes.^53^ Consequently, TGFBI blockade may represent a promising immunotherapeutic strategy, alone or in combination with other therapies, for tumors refractory to current mICIs, including MSS-MRR CRC and HCC (cold tumors). Furthermore, the enhancement of tumor-specific CD8^+^ CTLs on TGFBI blockade in PDO models may enable the development of adaptive cell therapies using autologous tumor-specific T cells, either directly or following genetically engineering to enhance antitumor activity. Finally, the increased frequency of memory and effector phenotype (CCR7^–^CD45RA^–^ or CCR7^–^CD45RA^+^) CD4^+^ or CD8^+^ T cells following TGFBI blockade can be used as a valuable biomarker to predict immunotherapy efficacy. The limitations of our study are that a larger cohort of patients is needed to support the role of TGFBI blockade in improving antitumor responses ex vivo and to investigate if TGFBI expression is modulated following ICI therapy, and that further analyses are required to explore whether TGFBI blockade could have a synergistic effect with current ICIs (ie, anti-PD-1 or anti-PDL-1), preliminarily by using autologous PDO-T cell co-culture systems. In addition, studies are required to investigate the transcriptional and epigenetic program allowing the T cell inhibitory effects by TGFBI, and to ascertain the inhibitory mechanisms on the differentiation and function of other TGFBI^+^ immune cells including monocytes, B cells, and NK cells in tumors.

## Data Availability

All data relevant to the study are included in the article or uploaded as supplementary information.
