# Supplementary material for: Transforming growth factor β induced as a novel secreted immune checkpoint counterinhibiting human tumor-associated T cells
Source: J Immunother Cancer. 2026 Apr 2;14(4):e012668. doi: 10.1136/jitc-2025-012668 (PMC13158596; doi:10.1136/jitc-2025-012668)
Supplement: Supplementary data [file jitc-14-4-s001.pdf]

Supplementary Material and Methods

ELISA assay

Serum samples and culture supernatant were diluted in the buffer supplied with the Human Transforming Growth Factor Beta induced protein ig-H3 (TGFBI/BIGH3) ELISA kit (Cusabio, Houston, USA) at 1:50 and 1:2, respectively. A standard curve, composed of 8 concentrations in ½ serial dilutions starting with 5000 pg/mL was prepared and run with serum and culture supernatant samples, all in duplicate. After stopping the colorimetric reaction, optical density was read at 450nm (Multiskan FC, Thermo Scientific) and concentrations were calculated by interpolation with the reference curve.

Isolation of TILs

Surgery-derived TUM and NTUM from CRC and HCC tissues were collected in 50mL falcon tubes pre-filled with culture medium (Gibco™ RPMI 1640 Medium) supplemented with 10% of penicillin (100 U/mL)/streptomycin (100 µg/mL) (EuroClone) and 10% of Amphotericin B (2.5 µg/mL) (Gibco™). Then, tissues were cut into small pieces, placed in 10 mL of HBSS (without Ca^{2+} and Mg^{2+}) 1mM of Dithiothreitol (DTT solution, Sigma-Aldrich) and incubated at 37°C for 15 minutes, followed by two washes in PBS-0.75mM EDTA (Sigma-Aldrich). After washing, tissue fragments were enzymatically dissociated in HBSS (with Ca^{2+} and with Mg^{2+}) supplemented with 2% of heat inactivated fetal bovine serum (FBS) (Gibco™), 6 mg/mL bovine serum albumin (BSA) (Sigma-Aldrich), 0.5 mg/mL collagenase IV (Sigma-Aldrich) and 50 ng/ml DNAase I (Roche) using gentleMACS Octo-Dissociator apparatus (Miltenyi Biotec Germany). Enzymatic reaction was stopped by adding cold HBSS (without Ca^{2+} and Mg^{2+}) and cell suspensions filtered through 70 µm filter into a 50mL falcon tube. After refilling, tubes with cold HBSS cells were spin for 10min at 1600rpm at RT. The supernatant was discarded by pipetting and the pellet resuspended in 5 mL of 40% Percoll in phosphate-buffered saline (PBS (1x), EuroClone S.p.A, Milan, Italy). After centrifugation at 1600 rpm for 10 min and discarded the supernatant, cell pellet

Rosado MM, *et al. J Immunother Cancer* 2026; 14:e012668. doi: 10.1136/jitc-2025-012668

was resuspended in 8 mL of RPMI 1640 medium supplemented with 10% heat-inactivated FBS, L-glutamine (2mM), penicillin (100 U/mL) and streptomycin (100 µg/mL), and sodium pyruvate (complete RPMI medium) and stratified in Ficoll (Lympholite®-H, Cedarlane, Southern Ontario, Canada). After centrifugation, cells were washed in PBS (1x) and stored at -80°c for future analysis.

Human Recombinant TGFBI treatment in vitro

PBMC from HDs (N=3) were seeded at a density of 0.2 x 10^6 cells per well with anti-CD3-CD28 beads (Invitrogen), at a ratio of 1:8, in DMEM supplemented with 10% heat inactivated FBS, 100 U/ml recombinant human IL-2 (5µg/mL, Roche, Germany) and recombinant human beta IG-H3 protein (R&D systems, Biotechne) at the concentration of 0.1µg/mL, 0.2 µg/mL, 0.4 µg/mL, 0.8 µg/mL and 1.6 µg/mL. Cells were left for 3 days at 37°C, 5% CO_2. 4hrs before harvesting, cells were incubated with Protein Transport Inhibitor Cocktail (brefeldin A and monensin, eBioscience™, Thermo Fisher Scientific). At the end of the stimulation cells were washed by centrifugation at 1200 rpm for 5 minutes at 4°C in PBS (1x) and stained for flow-cytometry analysis.

Tissue Microarray assay and Immunohistochemistry staining

Slides were deparaffinized at 72°C using Ventana EZ Prep reagent and hydrated, followed by an antigen retrieval method using Ventana Tris-EDTA buffer pH 7.8 for 64 minutes at 95°C. TGFBI protein was then detected using rabbit polyclonal anti-TGFBI (Sigma, HPA008612). Staining was visualized with chromogenic substrate 3,3′-diaminobenzidine (DAB) in association with UltraView Universal DAB detection Kit (Eurobio scientific, SK-4100) for colon and liver tissues. The sections were counterstained with Gill's Hematoxylin for 8 minutes and post counterstained with Bluing reagent for 4 minutes. Finally, the slides were washed in warm tap water with detergent and dehydrated in graded ethanol and methylcyclohexane, then coverslipped in permanent mounting media Pertex (MM France, F/00840). Images were analyzed using Halo-AI v3.6 software. Mininet algorithm was used

to discriminate tumoral and stromal areas. Multiplex algorithm was used to quantify positive signal. Samples were visually inspected to exclude incomplete sections (folded or detached from glass slides). TGFBI expression was quantified as percentage of stromal area.

Immunofluorescence on CRC and HCC tissues

First, sections were incubated with anti-TGFBI antibody overnight at 4°C, followed by 1-hour incubation at RT with Alexa Fluor-594 goat anti-rabbit IgG for the double IF with anti-EpCAM, anti-CD14 or anti-CD3, and with Alexa Fluor-488 for the double IF with anti-CD20. Next, slides were incubated overnight at 4°C either with Alexa Fluor-594 anti-CD20, Alexa Fluor-488 anti-CD3, or with the unconjugated anti-CD14 or anti-EpCAM antibodies. Stainings with anti-CD14 and anti-EpCAM were followed by 1-hour RT incubation with Alexa Fluor-488 goat anti-mouse IgG. Next, slides were counterstained for 5 minutes with Hoechst (H3570, Invitrogen) and cover-slipped with 60% glycerol in PBS. Confocal microscopy imaging was performed by Leica TCS-SP8Xlaser-scanning confocal microscope (Leica Microsystems) equipped with tunable white light laser source, 405 nm diode laser, 3 (PMT) e 2(HyD) internal spectral detector channels. Sequential confocal images were acquired using a HC PLAPO 40× oil immersion objective (1.30 numerical aperture, Leica Microsystems) with a 1024×1024 image format, scan speed 400Hz. Density of intra-tumoral T-cells, B-cells, monocytes, and epithelial cells expressing or not expressing TGFBI was recorded as number of positive cells per unit area (mm^2) by two blinded examiners, as previously described (36).

Supplementary figures and figure legends

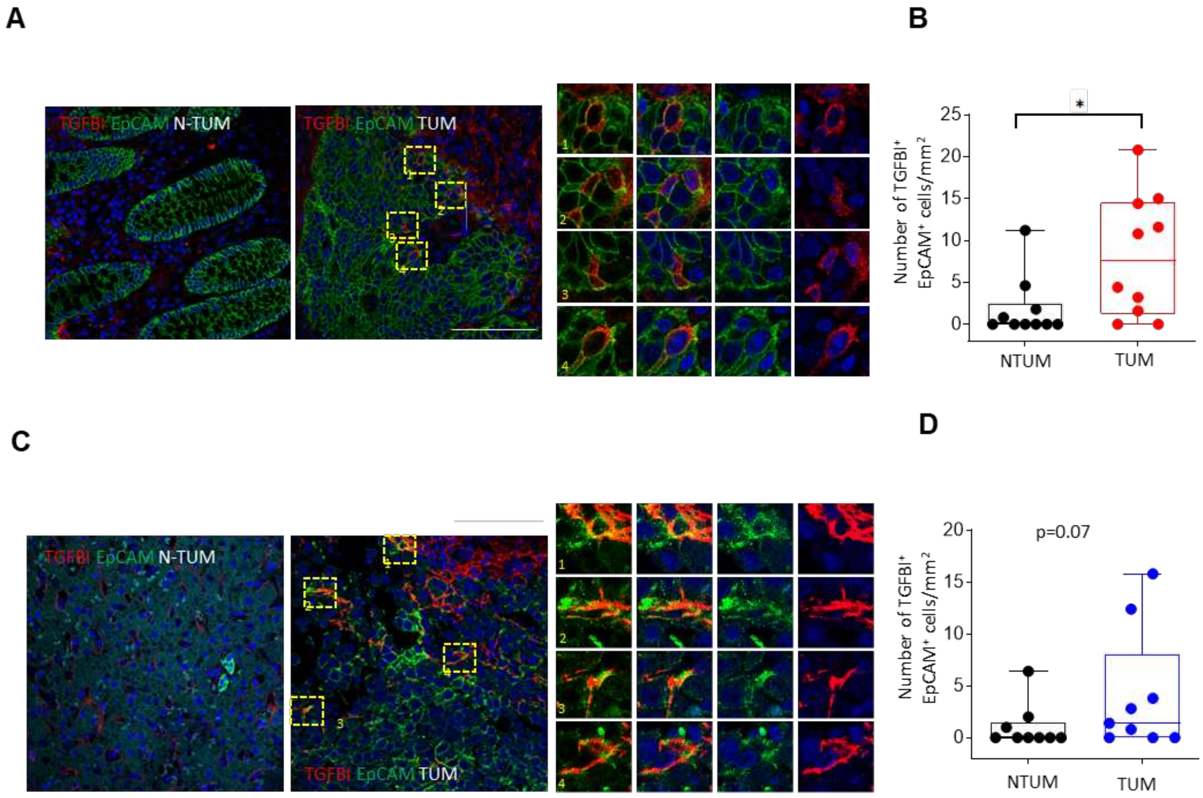

Supplementary Figure 1. TGFBI is overexpressed by EPCAM$^+$ TUM cells from CRC and HCC patients *in situ*.

Representative images from double co-staining for EpCAM$^+$ (green) and TGFBI$^+$ (red) cells from NTUM (left) and TUM (right) of one CRC (**A**) and one HCC (**C**) patient are shown (original magnification ×40, scale bar 30µm). Nuclei are counter-stained with Hoechst (blue). Four representative examples of double-expressing EpCAM$^+$TGFBI$^+$ cells within yellow rectangles are highly magnified on the bottom panels. Each panel shows merging of both fluorescent signals (EpCAM in green and TGFBI in red) without and with nuclei (blue) followed by individual staining. Quantitative analysis of the indicated cells from 10

Rosado MM, *et al. J Immunother Cancer* 2026; 14:e012668. doi: 10.1136/jitc-2025-012668

independent CRC (**B**) or 9 HCC (**C**) patients is shown. Levels of significance was determined by paired two-tailed Student's t-test. * *p <0.05.*

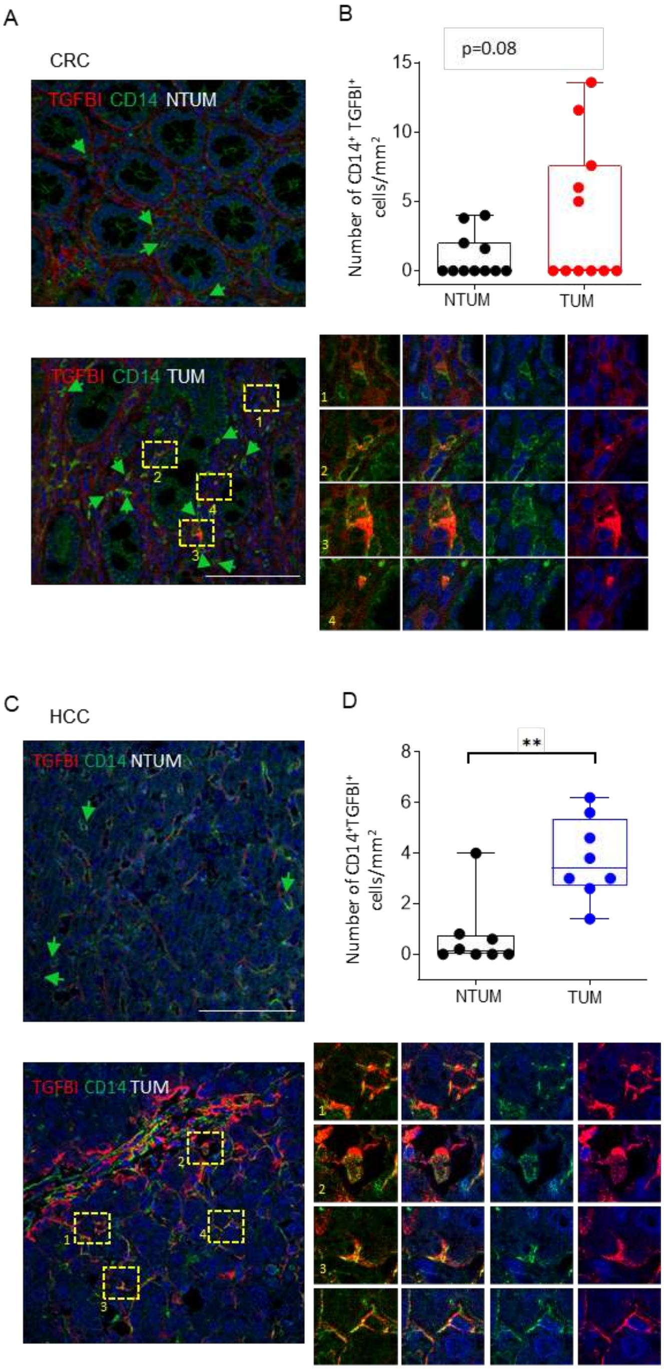

Supplementary Figure 2. TGFBI expression by CD14⁺ monocytes in TUM or NTUM areas from CRC and HCC patients*.*

Representative images from double co-staining of NTUM (top) and TUM (bottom) for $CD14^+$ (green) and $TGFBI^+$ (red) of a CRC (**A**) or a HCC (**C**) patient are shown (original magnification ×40, scale bar 30μm). Nuclei are counter-stained with Hoechst (blue). The green arrows indicate CD14-expressing immune cells. Four representative examples of double-positive $CD14^+TGFBI^+$ cells within yellow rectangles are highly magnified on the bottom panels. Each panel shows merging of both fluorescent signals (CD14 in green and TGFBI in red) without and with nuclei (blue) followed by individual staining. Quantitative analysis of the indicated cells from 10 independent CRC (**B**) or from 8 HCC (**D**) patients is shown. Levels of significance was determined by paired two-tailed Student's t-test. $**p<0.01$

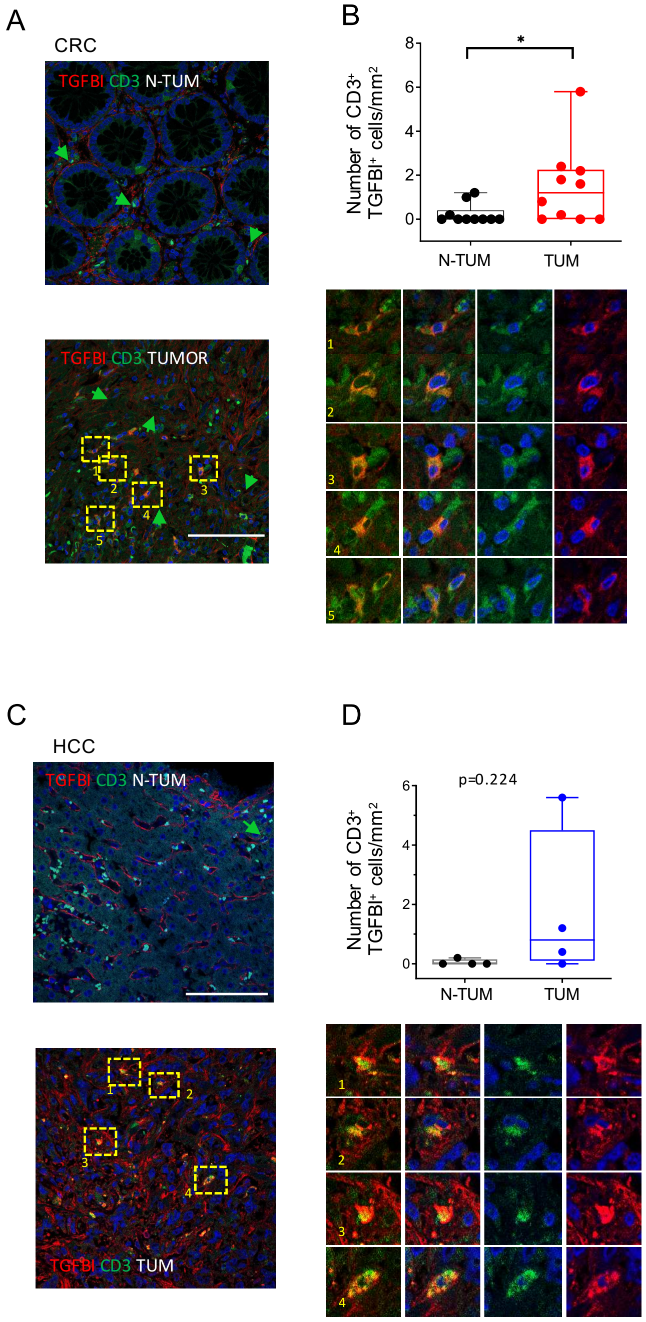

Supplementary Figure 3. TGFBI expression by CD3^{+} cells in TUM and NTUM areas from CRC and HCC patients.

Representative images from double co-staining of NTUM (top) and TUM (bottom) for CD3^{+} (green) and TGFBI^{+} (red) of a CRC (**A**) or a HCC (**C**) patient are shown (original

magnification ×40, scale bar 30μm). Nuclei are counter-stained with Hoechst (blue). The green arrows indicate CD3-expressing cells. Five representative examples of double-positive $CD3^{+}TGFBI^{+}$ cells within yellow rectangles are highly magnified on the bottom panels. Each panel shows merging of both fluorescent signals (CD3 in green and TGFBI in red) without and with nuclei (blue) followed by individual staining. Quantitative analysis of the indicated cells from 10 independent CRC (**C**) or from 4 HCC (**D**) patients is shown. Levels of significance were determined by paired two-tailed Student's t-test. * $p<0.05$

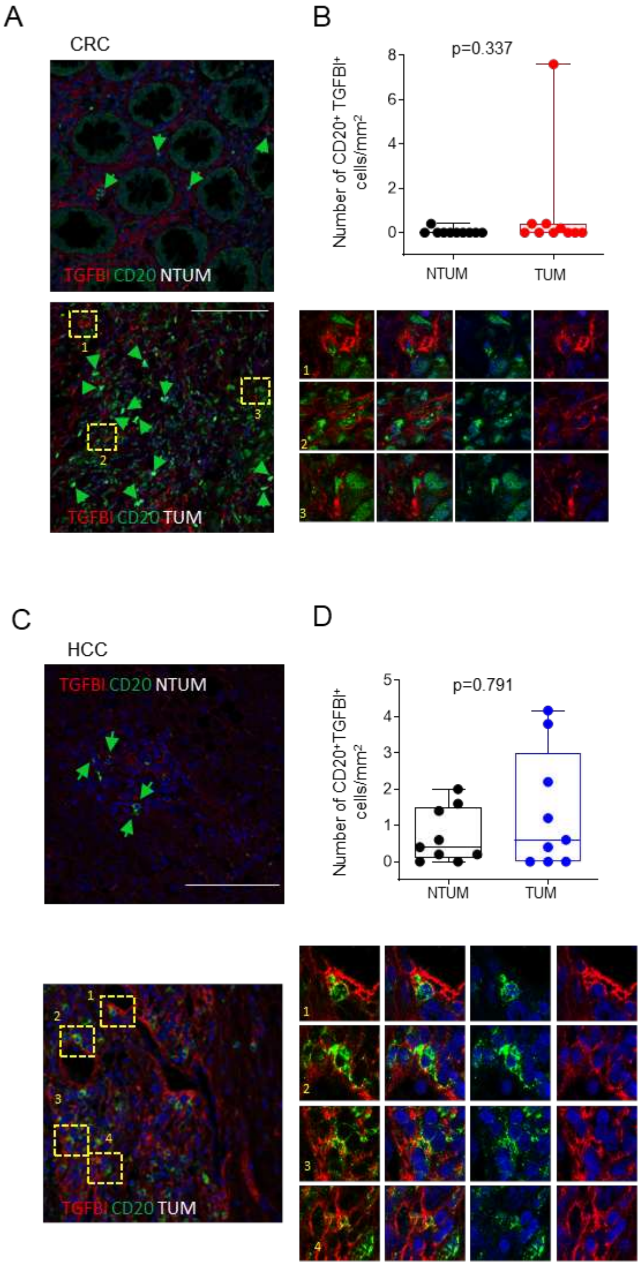

Supplementary Figure 4. TGFBI expression by CD20$^+$ cells in TUM or NTUM areas from CRC and HCC patients.

Representative images from double co-staining of NTUM (top) and TUM (bottom) for CD20$^+$ (green) and TGFBI$^+$ (red) of a CRC (**A**) or a HCC (**C**) patient are shown (original magnification ×40, scale bar 30µm). Nuclei are counter-stained with Hoechst (blue). The green arrows indicate CD20-expressing B cells. Three representative examples of double-

positive CD20^{+}TGFBI^{+} cells within yellow rectangles are highly magnified on the bottom panels. Each panel shows merging of both fluorescent signals (CD20 in green and TGFBI in red) without and with nuclei (blue) followed by individual staining. Quantitative analysis of the indicated cells from 10 independent CRC (**B**) or from 9 HCC (**D**) patients is shown. Levels of significance were determined by paired two-tailed Student's t-test.

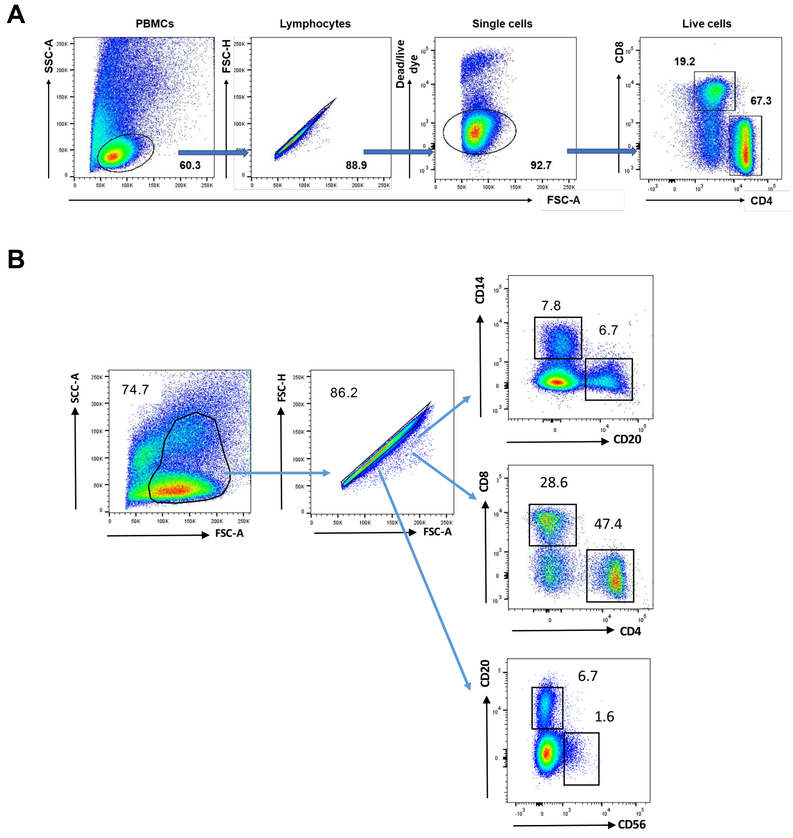

Supplementary Figure 5. Gating strategy of flow-cytometry analyses.

(**A**) Representative gating strategy used in the flow-cytometry analysis for all *in vitro* cultures. Cells were first identified has lymphocytes according to SSC-A, side scatter area; FSC-A, forward scatter area profile (first panel on the left) and has single cells with FSC-A

and FSC-H, forward scatter height (doublet exclusion). Single cells were selected for viability with LIVE/DEAD™ Fixable Near-IR, as negative cells and then subdivided into either $CD4^+$ or $CD8^+$ T cell populations. (**B**) Representative gating strategy used to detect intracellular TGFBI expression on $CD20^+$, $CD14^+$, $CD56^+$, $CD4^+$ and $CD8^+$ T cell subsets in peripheral blood and tissue infiltrating cells. Cells were first identified has monocyte/lymphocyte according to SSC-A/FSC-A profile (first panel on the left) and has single cells with FSC-A and FSC-H (doublet exclusion middle panel). $CD14^+$/$CD20^+$, $CD4^+$/$CD8^+$, and $CD20^+$/$CD56^+$ cell subsets are shown in the last three panels on the right.

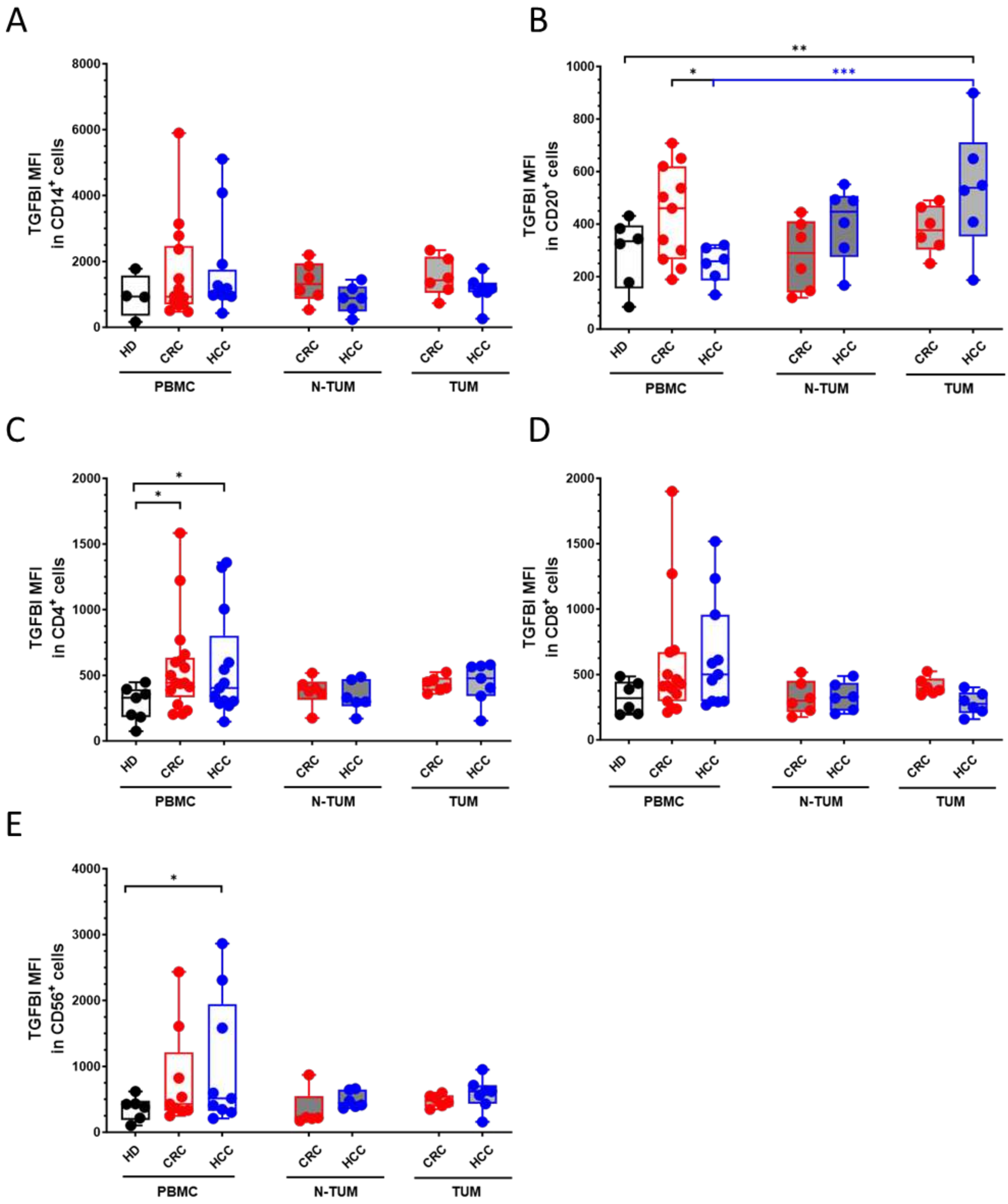

Supplementary Figure 6. Mean fluorescence intensity (MFI) in immune cell populations expressing TGFBI.

Box plots indicating median maximum and minimum mean fluorescence intensity (MFI) of TGFBI in monocytes (CD14$^+$) (**A**), T cells (CD4$^+$ or CD8$^+$) (**B, D**), B cells (CD20$^+$) (**C**), and

Rosado MM, *et al. J Immunother Cancer* 2026; 14:e012668. doi: 10.1136/jitc-2025-012668

NK cells ($CD56^+$) cells (**E**) in peripheral blood from HD (N=6), CRC (N=10) and HCC (N=10) patients, and normal tissue (N-TUM) / or tumor (TUM) infiltrating mononuclear cells from CRC (N=6) and HCC (N=6). * *p<0.05*, ***p<0.01,* *** *p<0.001* Ordinary one-way Anova using Tukey's multiple comparison tests and paired two-tailed Student's t-test to compared parameters between groups and samples from the same patient, respectively.

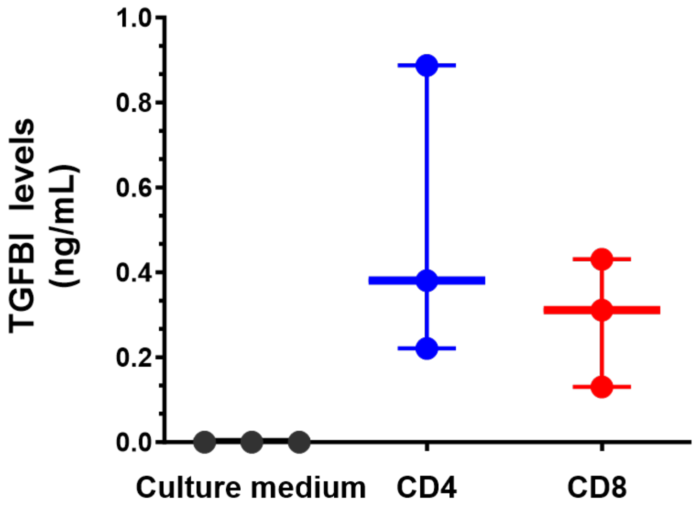

Supplementary Figure 7. TGFBI secretion by freshly purified CD4$^+$ and CD8$^+$ T cells. Levels of TGFBI (ng/mL) present in culture supernatants of freshly purified CD4$^+$ (blue) or CD8$^+$ (red) T cells. Data shows median maximum and minimum of 3 independent experiments.

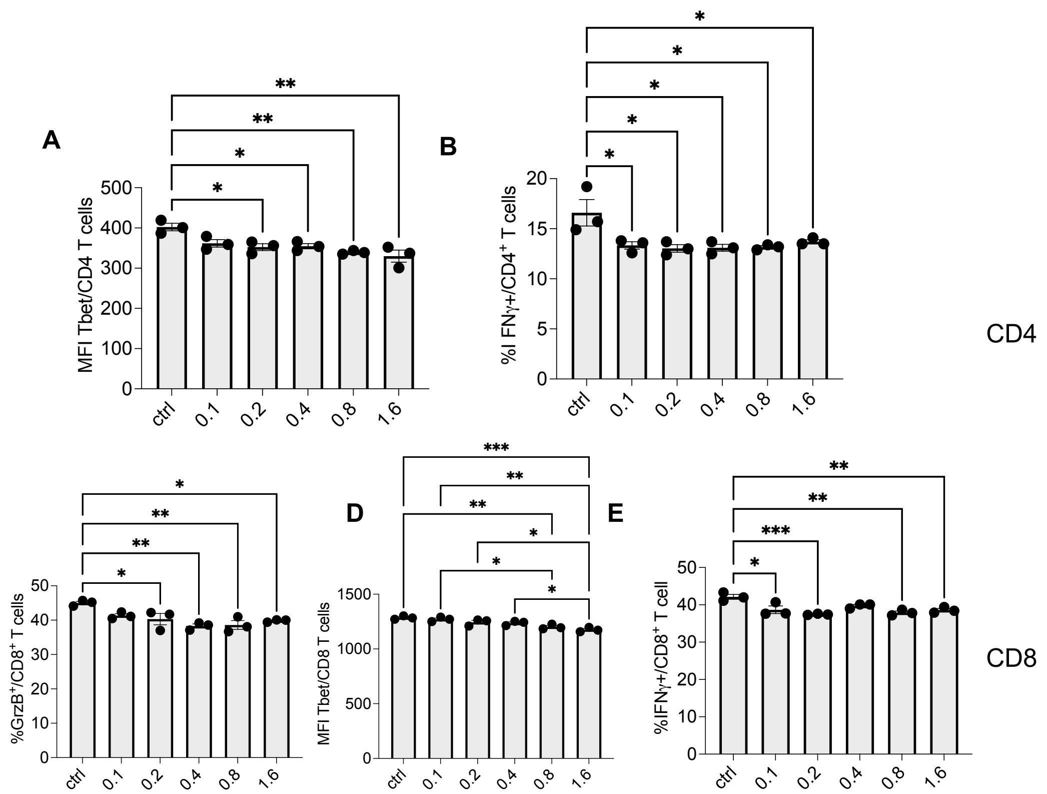

Supplementary Figure 8. TGFBI inhibits CD4+ and CD8+ T cell activation and function. PBMCs from 3 HDs were independently stimulated with anti-CD3/CD28 in the presence or absence (ctrl) of increasing concentrations of TGFBI recombinant (0,1- 0,2- 0,4- 0,8- 1,6 μg/mL) and then the expression of Tbet as MFI **(A)**, IFN-γ (as frequency) was evaluated in CD4+ (upper panels) **(B)** and GrzB and IFN-γ in CD8+ as frequency (bottom panels) **(C-E)** Tbet as MFI **(D)** T cells. One representative experiment is shown and represented in triplicate. * *p<0.05*, ***p<0.01, *** p<0.001* One-way Anova test using Tukey's multiple comparison tests.

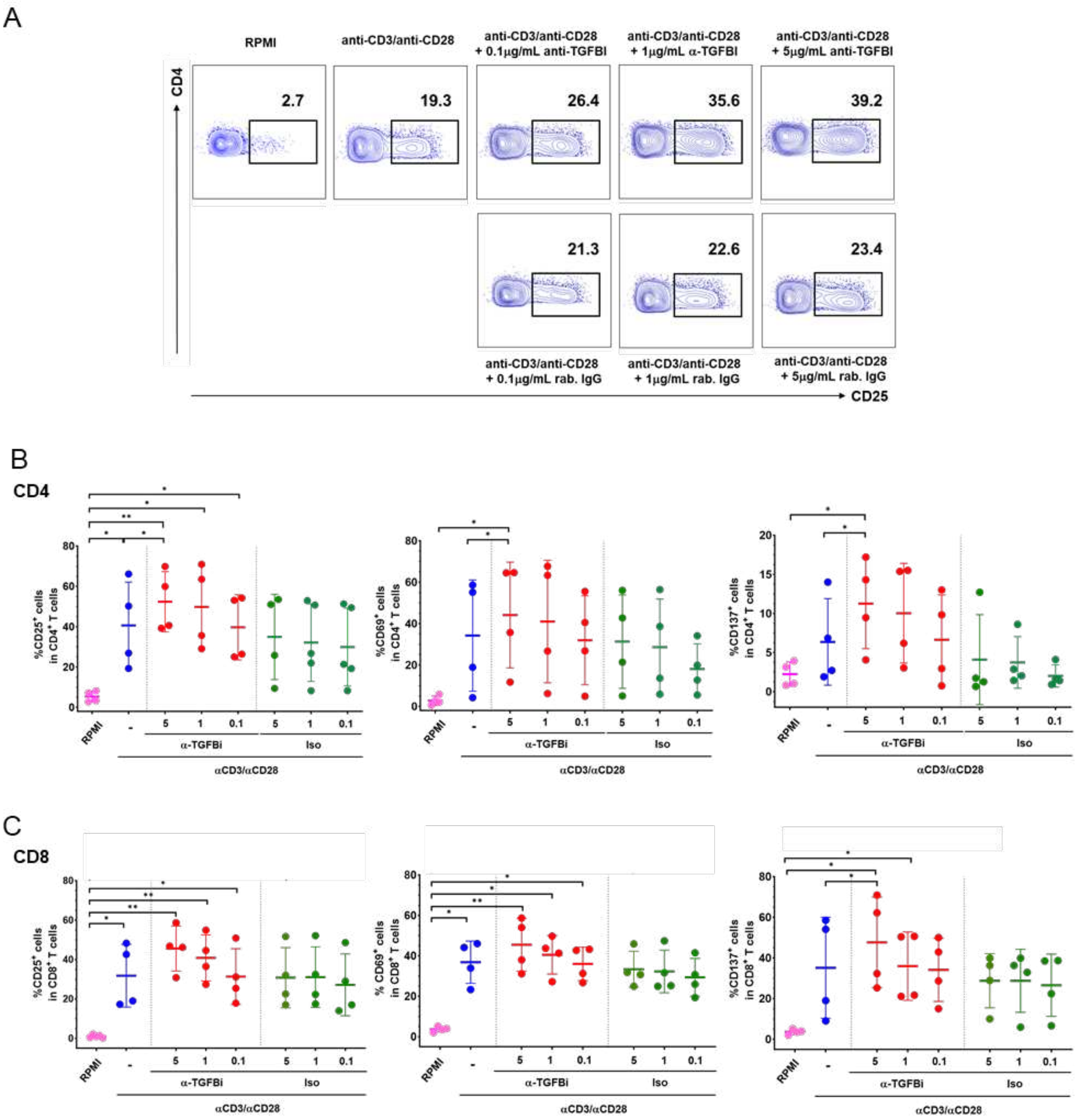

Supplementary Figure 9. Neutralization of TGFBI by anti-TGFBI antibody.

(**A**) Representative flow-cytometry contour plot analysis, with frequencies, of non-stimulated (RPMI) or anti-CD3/CD28-stimulated CD25$^+$ cells gated in CD4$^+$ T cells in PBMCs from a CRC patient, in the presence or absence of increasing concentrations of neutralizing anti-TGFBI antibody (0,1-1-5 µg/mL) or the corresponding isotype control. (**B-C**) Box plots indicating median maximum and minimum frequencies of non-stimulated or anti-CD3/CD28-stimulated CD25$^+$, CD69$^+$, CD137$^+$ cells gated in CD4$^+$ (upper panels) or CD8$^+$ (lower

panels) T cells in PBMCs from CRC patients (N=4) in the presence or absence of increasing concentrations of neutralizing anti-TGFBI antibody or the corresponding isotype control. Significance was determined by paired two-tailed Student's t-test between RPMI and stimulated cells, and within different stimulation conditions, for single CRC patients. *$p<0.05$, **$p<0.01$*.

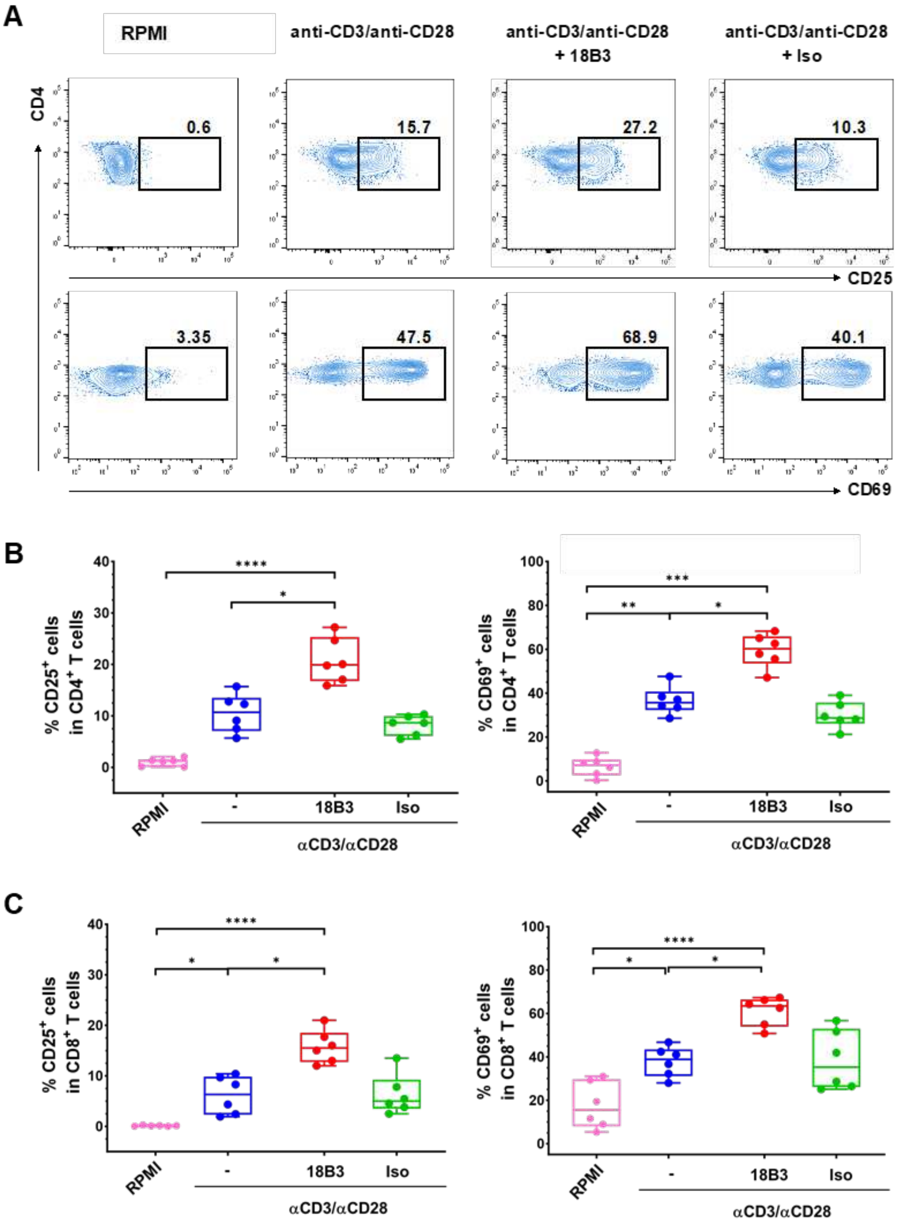

Supplementary Figure 10. Neutralization of TGFBI by anti-TGFBI (18B3) antibody.

(**A**) Representative flow-cytometry contour plot analysis, with frequencies, of non-stimulated (RPMI) or anti-CD3/CD28-stimulated CD25+ and CD69+ cells gated in CD4+ T cells in PBMCs from HD, in the presence or absence of neutralizing anti-TGFBI antibody (18B3 at 3μg/mL) or the corresponding isotype control. (**B-C**) Box plots indicating median maximum and minimum frequencies of NS or anti-CD3/CD28-stimulated CD25+, CD69+ cells gated in

$CD4^+$ (B) or $CD8^+$ (C) T cells in PBMCs from HDs (N=6) in the presence or absence of 18B3 neutralizing anti-TGFBI antibody or the corresponding isotype control. Significance was determined by paired two-tailed Student's t-test between RPMI and stimulated cells, and within different stimulation conditions, for single HDs. * $p<0.05$, ** $p<0.01$, *** $p<0.001$ and **** $p<0.0001$

Rosado MM, *et al. J Immunother Cancer* 2026; 14:e012668. doi: 10.1136/jitc-2025-012668

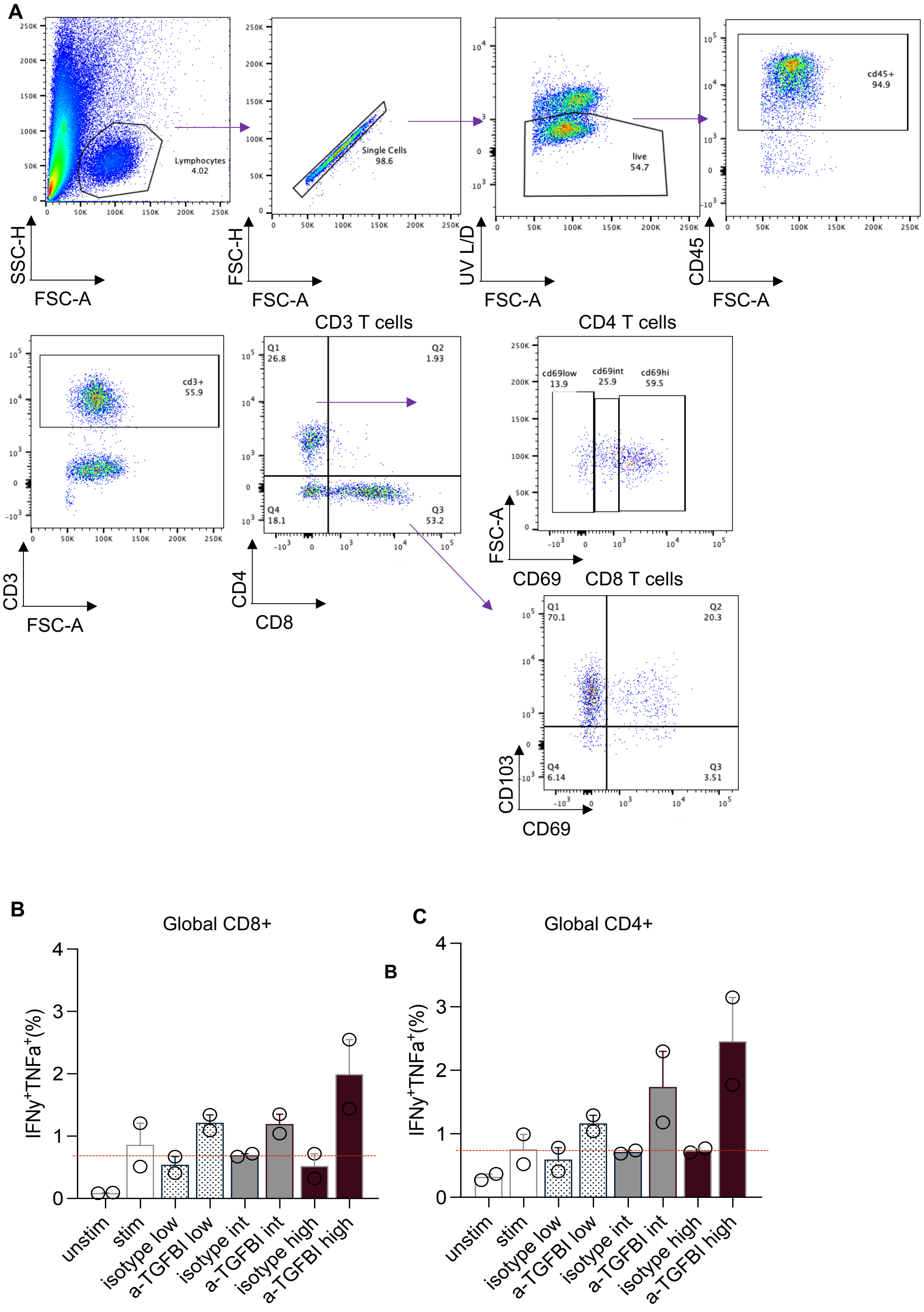
A
Lymphocytes
4.02
Single Cells
98.6
live
54.7
cd45+
94.9
SSC-H
FSC-A
FSC-H
FSC-A
UV L/D
FSC-A
CD45
FSC-A
cd3+
55.9
CD3
FSC-A
CD3 T cells
Q1
26.8
Q2
1.93
Q4
18.1
Q3
53.2
CD4
CD8
CD4 T cells
cd69low
13.9
cd69int
25.9
cd69hi
59.5
FSC-A
CD69
CD8 T cells
Q1
70.1
Q2
20.3
Q4
6.14
Q3
3.51
CD103
CD69
B
Global CD8+
IFNy+TNFa+(%)
unstim
stim
isotype low
a-TGFBI low
isotype int
a-TGFBI int
isotype high
a-TGFBI high
C
B
Global CD4+
IFNy+TNFa+(%)
unstim
stim
isotype low
a-TGFBI low
isotype int
a-TGFBI int
isotype high
a-TGFBI high

Supplementary Figure 11. Gating strategy and comparison of global intrahepatic CD8$^+$ and CD4$^+$ T cell responses to stimulation in the presence of TGBI blocking antibody or a matched isotype control at 3 concentrations.

(A) Representative flow-cytometry dot plot showing gating strategy of CD69$^{high/dim/low}$ CD4$^+$ T cells and CD103/CD69 CD8$^+$ T cells in intrahepatic CD3$^+$ lymphocytes. **(B-C)** Intrahepatic lymphocytes isolated from non-tumor associated liver tissues were unstimulated or anti-CD3/CD28-stimulated in the presence or absence of the neutralizing anti-TGFBI antibody or isotype at high- 3.0 μg/mL, intermediate- 1.0 μg/mL and low- 0.3 μg/mL concentrations, overnight and analyzed by flow cytometry. Dotted red line indicates threshold frequency of IFNγ$^+$TNFα$^+$ cells with isotype control blockade (N=2).

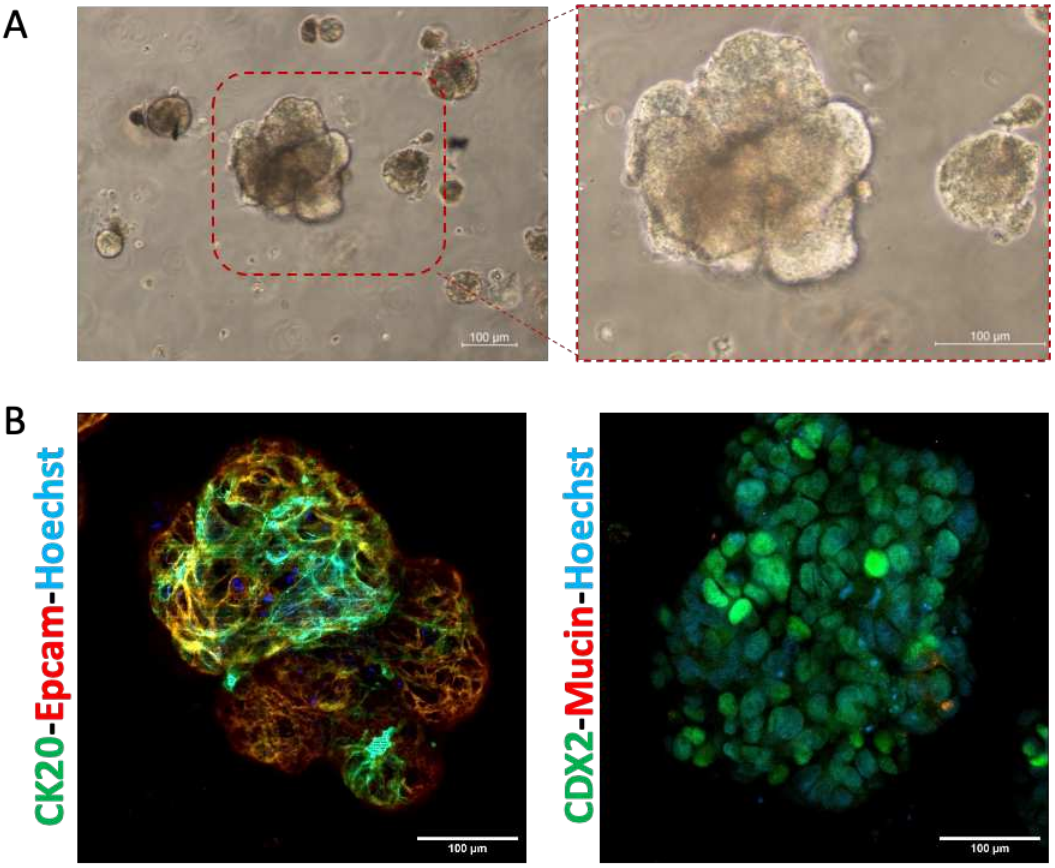

Supplementary Figure 12. Characterization of human PDOs.

(**A**) Morphological analysis of a representative human CRC organoid with bright-field images. (**B**) Confocal images of immunofluorescence (IF) staining for colorectal cancer markers (CDX2, MUC1, CK20, EpCAM) and Hoechst (DNA), as indicated. A representative PDO is shown. Scale bar: 100 µm.

Rosado MM, *et al. J Immunother Cancer* 2026; 14:e012668. doi: 10.1136/jitc-2025-012668

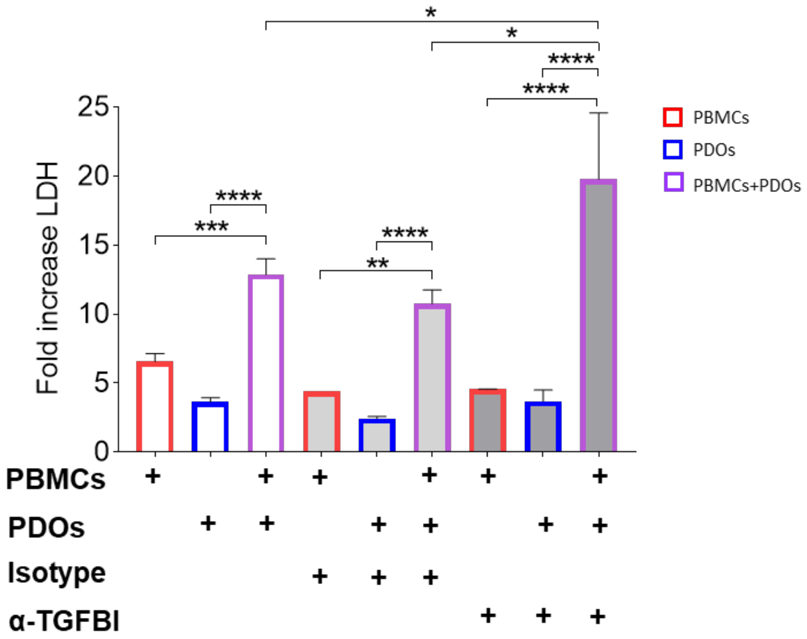

Supplementary Figure 13. Effect of different concentrations of neutralizing anti-TGFBI antibody on PDO cell killing.

(A) HD PBMCs (red bar) and PDO cells (blue bar) alone or in co-culture (violet bar) at an effector (E): target (T) ratio of 20:1, in the presence (grey-filled bars) or absence (empty bars) of neutralizing anti-TGFBI antibody or the corresponding isotype (Iso) control (open bars) at 10μg/mL. Target cell killing was assessed after 72h of culture by quantification of LDH released. One representative experiment is shown. * p<0.05, **p<0.01, *** p<0.001 and **** p < 0.0001 by one-way Anova.

Rosado MM, *et al. J Immunother Cancer* 2026; 14:e012668. doi: 10.1136/jitc-2025-012668

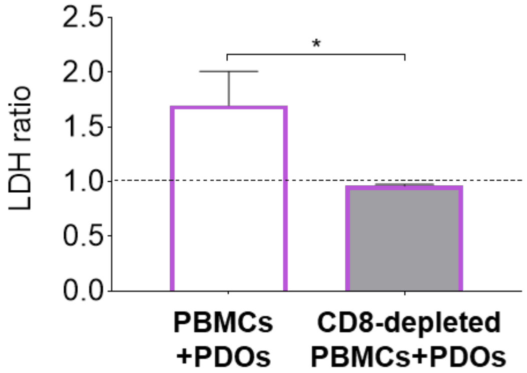

Supplementary Figure 14. PDO killing by CD8⁺ T cell depleted PBMCs.

PDOs were co-cultured for 72 h either with total PBMCs (PDO+PBMC) or with CD8⁺ T cell–depleted PBMCs (PDO+CD8 dep PBMC). Cytotoxicity was quantified by measuring LDH release in culture supernatants. LDH values were normalised to LDH baseline values of both effectors (PBMCs) and targets (PDOs). Spontaneous LDH release by PDOs alone is set as 1, (dashed line). Bars represent mean ± SEM of 2 independent experiments. Statistical analysis was performed unpaired two-tailed Student's t-test. * $p < 0.05$

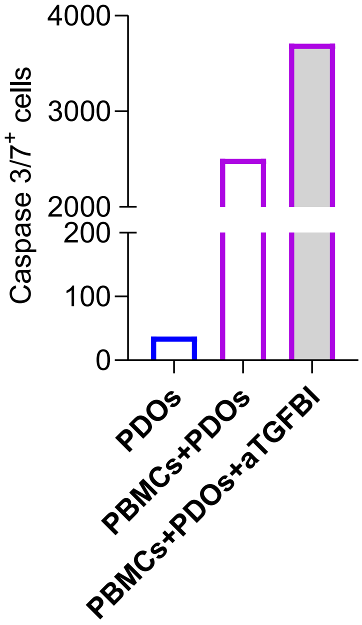

Supplementary Figure 15. PDO killing by PBMCs increases with TGFBI neutratization

HD PBMCs and PDOs were cultured alone or in co-culture, with or without the neutralizing anti-TGFBI antibody (10 µg/mL). PBMCs were stained with CMPTX (red) and PDOs with Caspase 3/7 dye (green). For Caspase 3/7 quantification, green-positive cells were counted with QuPath software; the sum of green-positive cells in four fields acquired at 10X magnification, for each sample, is represented.

Supplementary Table 1. Antibodies used for IF analysis

Marker	Fluorochrome	Clone	Company	Catalogue number	***RRID index
TGFBI	-	EPR12078(B)	Abcam	ab170874	AB_2895231
CD3	Alexa Fluor488	UCHT1	BioLegend	300415	AB_389310
CD14	-	1H5D8]	abcam	ab181470	AB_2725781
CD20	Alexa Fluor594	2H7	BioLegend	302354	AB_2565619
EpCAM	-	VU1D9	Cell Signaling	2929	AB_2098657
CDX2	-	D11D10	Cell Signaling	12306	AB_2797879
Keratin 20	-	D9Z1ZXP®	Cell Signaling	13063	AB_2798106
MUC1		VU4H5	Cell Signaling	4538	AB_2148549
Goat-αmIgG*	Alexa Fluor488	-	Thermo Fisher Scientific	A-11017	AB_143160
Goat-αrIgG**	Alexa Fluor594	-	Thermo Fisher Scientific	A-11037	AB_2534095

* goat anti-mouse IgG (H+L) secondary antibody

** goat anti-rabbit IgG (H+L) secondary antibody

*** RRID= Research Resource Identifier

Supplementary Table 2. Antibodies used for flow cytometry (FACS) analysis on human samples.

Antigen (human)	Flurochrome	Clone	Company	Catalogue number	*RRID index
CD4	**BV711	RM4-5	BD Biosciences	563726	AB_2738389
CD4	BV785	OKT4	Biolegend	317442	AB_2563242
CD8	BV510	SK1	Biolegend	344732	AB_2564624
CD8	Pacific Blue	HIT8a	Biolegend	300928	AB_10612929
CD14	eFluor780	61D3	Invitrogen	47014942	AB_1834358
CD20	BV785	2H7	BD Biosciences	743611	AB_2741622
CD25	APC	BC96	BD Biosciences	555434	AB_398598
CD45RA	BV605	HI100	Biolegend	304134	AB_2563814
CD56	PerCP/Cy5.5	MEM188	Biolegend	304625	AB_10639946
CD69	AF700	FN50	Biolegend	310922	AB_493775
CD103	BV 711	ber-act8	BioLegend	350222	AB_2629651
CD107a	PE	H4A3	BD Biosciences	555801	AB_396135
CD137	PE/Dazzle 594	4B4-1	Biolegend	309826	AB_2566260
CD197	PerCP/Cy5.5	G043H7	Biolegend	353220	AB_10916121
Granzyme B	BV 510	GBII	BD Biosciences	563388	AB_2738174
IFN-γ	BV 711	4S.B3	Biolegend	502540	AB_2563506
TNF-α	BV 421	MAb11	Biolegend	502932	AB_10960738
TGFBI/bIGH3-rabbit poly. (IgG)	CoraLite 488	-	Proteintech	CL488-10188	AB_2918960

* RRID= Research Resource Identifier; ** BV= Brilliant Violet
